# Field experiments underestimate aboveground biomass response to drought

**DOI:** 10.1038/s41559-022-01685-3

**Published:** 2022-03-10

**Authors:** György Kröel-Dulay, Andrea Mojzes, Katalin Szitár, Michael Bahn, Péter Batáry, Claus Beier, Mark Bilton, Hans J. De Boeck, Jeffrey S. Dukes, Marc Estiarte, Petr Holub, Anke Jentsch, Inger Kappel Schmidt, Juergen Kreyling, Sabine Reinsch, Klaus Steenberg Larsen, Marcelo Sternberg, Katja Tielbörger, Albert Tietema, Sara Vicca, Josep Peñuelas

**Affiliations:** 1 Institute of Ecology and Botany, Centre for Ecological Research, Vácrátót, Hungary; 2 ‘Lendület’ Landscape and Conservation Ecology, Institute of Ecology and Botany, Centre for Ecological Research, Vácrátót, Hungary; 3 Department of Ecology, University of Innsbruck, Innsbruck, Austria; 4 Department of Geosciences and Natural Resource Management, University of Copenhagen, Frederiksberg, Denmark; 5 Namibia University of Science and Technology, Windhoek, Namibia; 6 Plants and Ecosystems (PLECO), Department of Biology, University of Antwerp, Wilrijk, Belgium; 7 Department of Forestry and Natural Resources, Purdue University, West Lafayette, IN USA; 8 Department of Biological Sciences, Purdue University, West Lafayette, IN USA; 9 CSIC, Global Ecology Unit CREAF-CSIC-UAB, Bellaterra, Spain; 10 CREAF, Cerdanyola del Vallès, Spain; 11 Global Change Research Institute of the Czech Academy of Sciences, Brno, Czech Republic; 12 Disturbance Ecology, Bayreuth Center of Ecology and Environmental Research, University of Bayreuth, Bayreuth, Germany; 13 Experimental Plant Ecology, University of Greifswald, Greifswald, Germany; 14 UK Centre for Ecology & Hydrology, Bangor, UK; 15 School of Plant Sciences and Food Security, Faculty of Life Sciences, Tel Aviv University, Tel Aviv, Israel; 16 Plant Ecology Group, University of Tübingen, Tübingen, Germany; 17 Institute for Biodiversity and Ecosystem Dynamics (IBED), Ecosystem and Landscape Dynamics (ELD), University of Amsterdam, Amsterdam, the Netherlands

**Keywords:** Ecosystem ecology, Climate-change ecology

## Abstract

Researchers use both experiments and observations to study the impacts of climate change on ecosystems, but results from these contrasting approaches have not been systematically compared for droughts. Using a meta-analysis and accounting for potential confounding factors, we demonstrate that aboveground biomass responded only about half as much to experimentally imposed drought events as to natural droughts. Our findings indicate that experimental results may underestimate climate change impacts and highlight the need to integrate results across approaches.

## Abstract (web summary)

In a meta-analysis comparing experimental versus observational studies of aboveground biomass responses to drought in grasslands, the authors show that effect sizes in experiments are 53% weaker than in observational studies, suggesting that experiments are underestimating drought responses.

## Main

To assess how climatic changes will affect ecosystems, field researchers commonly use one of two approaches: in situ observations or manipulative experiments. Observations have the advantage of being able to cover large areas and long time periods, but the links between ecosystem processes and climatic conditions are only correlational. In contrast, experiments can directly test responses to a given factor (for example, a manipulated climate variable) and isolate the effects of individual factors that often correlate with others in real-world settings. But experiments face logistical limits to their size and duration, and manipulated variables may poorly mimic natural changes or cause unwanted side effects^1,2^. Despite the differences between experiments and observations, few data syntheses compare the two types of studies. A recent overview of ecological responses to global change^3^ found that an overwhelming majority of meta-analyses covered either experimental or observational case studies, while only 3 out of 36 assessed both types. Furthermore, global estimates of ecosystem functioning have been based on upscaling from either experiments^4^ or observations^5^, but not both. The shortage of cross-domain syntheses is particularly remarkable because some comparisons have reported clear differences in results from the two approaches^6^.

In the coming decades, drought frequency and severity are projected to increase in many regions^7,8^. Droughts affect ecosystem functioning, including processes that influence climate^9^ (for example, carbon sequestration and transpiration). Although many observational and experimental studies have assessed the effects of drought events, no synthesis study on droughts has compared results from these two approaches (but see ref. ^10^ for a single-site comparison). A recent review identified 564 papers studying ecological effects of droughts in the past 50 years^11^; the majority of studies were observational. In contrast, reviews and meta-analyses of drought effects on net primary production (NPP) or aboveground biomass (AGB) focused almost exclusively on experiments, with only a single synthesis paper covering (but not comparing) both experimental and observational studies (Supplementary Note 1). This bias towards experimental drought studies is concerning in light of the limitations of climate change experiments, such as small spatial extent^2^ and inability to replicate the full set of naturally occurring drought conditions^1^.

We compared responses of AGB to experimentally applied versus observed drought events in a systematic review using hierarchical meta-analyses. We tested for effects of potential confounding factors such as drought severity (per cent reduction in annual precipitation), drought length (years) and site aridity (the ratio of mean annual precipitation (MAP) to mean annual potential evapotranspiration (PET), MAP/PET). We first identified studies that (1) were conducted in grasslands or shrublands, (2) were conducted in natural or semi-natural systems in the field, and (3) reported aboveground NPP (ANPP), AGB or plant cover. We then excluded from our focal analysis studies from wet sites or shrublands or that estimated plant cover, because these were rare and very unequally distributed between experiments and observations. Our focal analysis included 158 data points (75 experimental and 83 observational) from 80 studies (40 experimental, 39 observational and 1 that included both types). Drought plots were compared with control plots in the experimental studies, and drought years were compared with control (non-drought) years in the observational studies. In our focal meta-analysis, we weighted the data by the number of replications. We also conducted additional meta-analyses with different weightings, and using the data that were excluded from the focal analysis, to test the robustness of our results.

**Fig. 1:**
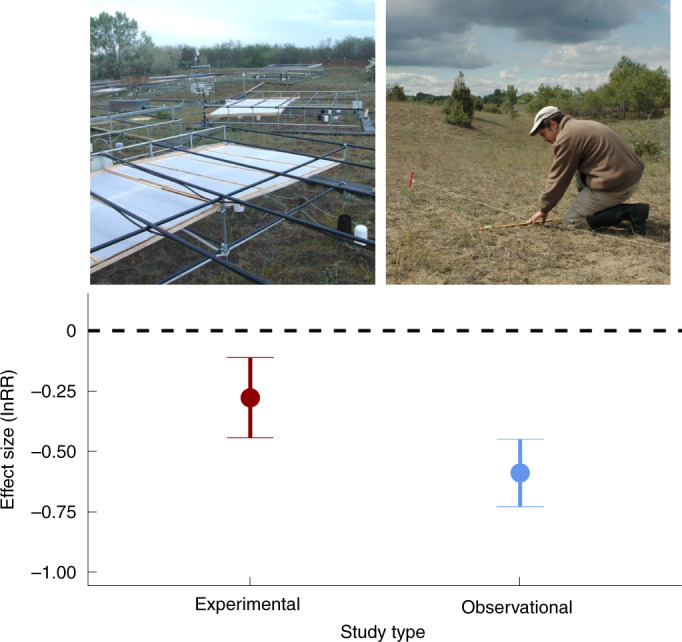
Response of aboveground biomass to drought measured by lnRR in experimental and observational studies in the focal meta-analysis. The results are model estimates from a meta-analytical model (Supplementary Note 2), presented as mean ± 95% CI (*n* = 75 for experiments and *n* = 83 for observations). The pictures show a drought experiment (left) and an observational study (right), both in the sand grasslands of central Hungary. (Photos by G.K.-D.)

**Fig. 2:**
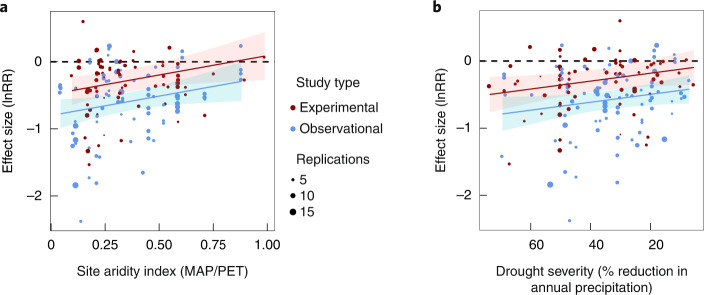
Responses of aboveground biomass to drought in experimental and observational studies as functions of site aridity and drought severity. **a**,**b**, The lines depict relationships between lnRR and site aridity index (AI) (**a**) and drought severity (**b**) modelled using a meta-analytical model (Supplementary Note 2), and the shaded bands show 95% CIs (*n* = 75 for experiments (red) and *n* = 83 for observations (blue)). AI was measured as MAP/PET; note that larger numbers indicate lower aridity, and 1 indicates that MAP equals PET. Drought severity was calculated as the per cent reduction in annual precipitation in drought plots (drought years in observational studies) compared with control plots (years). The circle sizes are proportional to the number of replications in the studies, which was used as a weighting factor in the meta-analysis. For the test results, see Supplementary Note 2.

The estimated mean effect of drought was 53% (95% confidence interval (CI), 16% to 90%) weaker in experimental than in observational studies, after controlling for potentially confounding factors (Fig. 1 and Supplementary Note 2). Drought responses increased with increasing aridity and marginally with increasing drought severity (Fig. 2 and Supplementary Note 2) but were not significantly affected by drought length (Supplementary Note 2). Interactions between study type and the other variables (site aridity, drought severity and drought length) were not significant, so we conclude that drought responses were stronger in observational than in experimental studies irrespective of site aridity and drought severity.

The results were very similar when we conducted an additional, variance-weighted meta-analysis on a subset of data with available estimates of variance: responses were weaker in experimental studies, at less arid sites and in less severe droughts (Supplementary Note 3). Furthermore, the response of AGB to drought was weaker in experiments than in observations when we conducted an unweighted meta-analysis (marginal significance; Supplementary Note 4) or analysed the data that were excluded from the focal analysis (wet sites, grasslands with plant cover data and shrublands; Supplementary Note 5). This latter finding suggests that the general pattern of weaker response in experiments holds beyond grasslands (focal dataset), even if the low number and unequal distribution of studies did not allow for a detailed analysis across a broader range of ecosystems.

The mean response to drought that we found for experiments (natural logarithm of the response ratio (lnRR), −0.28; Fig. 1) was similar to previous meta-analyses of drought experiments (lnRR, −0.2 to −0.28; refs. ^12–14^), indicating that the difference between experimental and observational studies was not due to a weaker response in experiments than in previous studies. Also, for our focal dataset, site aridity, drought severity and AGB (control) were similar in experimental and observational studies, and droughts lasted longer in experimental than in observational studies (Supplementary Note 6), so these factors seem unlikely to explain the weaker drought response of AGB in experiments than in observations. Publication bias was not detected for data included in the focal meta-analysis (Supplementary Note 7) and was therefore not considered to account for the large difference in response.

Our findings suggest that experiments considerably underestimate the effects of droughts in grasslands and shrublands. This discrepancy may occur in part because experiments typically cover small areas, and conditions in the surrounding landscape may dilute the intended treatment severity (creating an ‘island effect’^1,2^). Although we did not find a relationship between the size of drought experiments and the effect size of AGB response to drought in our focal dataset (Supplementary Note 8), even the largest experiments (few studies were >100 m^2^) were much smaller than the spatial extent of natural drought events. Note that the island effect may also sometimes strengthen the treatment effect in experiments, but this usually happens as a secondary effect due to altered primary production or species composition (such as congregation or avoidance of animals^15^). A difference between experiments and observational studies could also arise from differences in drought severity. It has been suggested that experiments tend to exaggerate drought severity relative to natural droughts^16^. However, we found that drought severity was similar across experimental and observational studies, and we used an analysis that accounted for drought severity. A potential reason for the underestimation of drought effects in experiments could be that they simulate less rain but do not control for increased evaporative demand associated with high temperatures, low humidity and clear skies. Given that droughts in reality are typically accompanied by these intensifying factors^17^, we assert that drought experiments underestimate drought effects as manifested in nature, rather than that observational studies overestimate them. In practice, using a drought severity metric that incorporates not only precipitation reduction but also variables such as temperature, humidity and cloud cover could narrow the gap between experimental and observational results. However, infrequent reporting of these variables in individual studies hinders such analyses^11^. Nevertheless, our findings that experimental and observational studies reported similar responses to changing site aridity and to changing drought severity suggest that experiments capture the major patterns of drought effects while underestimating the magnitude of the effects.

Reviews rarely compare the effects of environmental changes across study types, but from the existing comparisons, a consistent pattern emerges. Compared with experimental studies, observational studies have reported stronger effects of warming on plant phenology^6^, of fire on soil microbial biomass^18^, of disturbance on non-native plants^19^, of biological invasions on species richness^20^ and of fragmentation on insect abundance^21^. Mechanisms suggested for these patterns were the same as those that may explain the differential drought effects in our study—namely, the small spatial extent^21^ and incomplete representation of environmental change factors in experiments^18,20^. Further work is needed to test the generality of the observed discrepancies between experimental and observational results, and this should include both systematic comparison of study types across global change factors and matched case studies, where observational and experimental results come from the same sites. Yet, the common pattern across a wide range of environmental change factors listed above suggests that ecosystem manipulations, in general, tend to report weaker responses than observational studies.

Experiments have unique value even if they underestimate ecosystem responses to environmental change. Observational studies lack true controls, so observed relationships between processes and drivers are only correlational. When driving variables are correlated, as often happens in nature, the effects of individual drivers are difficult to disentangle; thus, observational studies provide limited understanding of underlying mechanisms^1^. Observations and experiments should each be used for their strengths: observations to estimate the ‘real’ net effects of climate change in realistic settings including all interacting factors, and experiments to test causation for clearly defined and experimentally reproducible driving variables and thereby obtain a mechanistic understanding. This is nicely exemplified in studies of warming effects on phenology: although warming experiments have been shown to dramatically underestimate phenological responses to warming^6^, experiments are still of great value for separating the relative effects of different factors on phenological changes in an era of warming^22^. Most importantly, our results emphasize the need to integrate results from different approaches instead of focusing on one approach and overlooking others, as seems to be common for studies of drought effects on AGB (Supplementary Note 1).

Reliable estimates of the magnitude of ecosystem responses to a changing climate are critically important when they are used for deriving broad-scale, sometimes global, estimates of potential change. Our results, together with those of other studies that indicate smaller responses in experimental settings than in observational studies, suggest caution when such estimates are based solely on experiments, such as when estimating change in the global stock of soil carbon on the basis of warming experiments^4^, change in global AGB on the basis of CO_2_-enrichment experiments^23^ or the responses of net ecosystem exchange to changes in precipitation on the basis of precipitation experiments^24^.

We conclude that while ecosystem experiments are an invaluable tool for studying the impacts of climate change, especially to distinguish among the effects of factors that change simultaneously and to unravel the mechanisms of ecosystem responses, they may underestimate the magnitude of the effects of climate change. Thus, innovative new work that integrates experimental and observational datasets could more reliably quantify the effects of climate change on terrestrial ecosystems.

## Methods

### Literature search and study selection

A systematic literature search was conducted in the ISI Web of Science database for observational and experimental studies published from 1975 to 13 January 2020 using the following search terms: TOPIC: (grassland* OR prairie* OR steppe* OR shrubland* OR scrubland* OR bushland*) AND TOPIC: (drought* OR ‘dry period*’ OR ‘dry condition*’ OR ‘dry year*’ OR ‘dry spell*’) AND TOPIC: (product* OR biomass OR cover OR abundance* OR phytomass). The search was refined to include the subject categories Ecology, Environmental Sciences, Plant Sciences, Biodiversity Conservation, Multidisciplinary Sciences and Biology, and the document types Article, Review and Letter. This yielded a total of 2,187 peer-reviewed papers (Supplementary Fig. 1). At first, these papers were screened by title and abstract, which resulted in 197 potentially relevant full-text articles. We then examined the full text of these papers for eligibility and selected 87 studies (43 experimental, 43 observational and 1 that included both types) on the basis of the following criteria:

1. The research was conducted in the field, in natural or semi-natural grasslands or shrublands (for example, artificially constructed (seeded or planted) plant communities or studies using monolith transplants were excluded). We used this restriction because most reports on observational droughts are from intact ecosystems, and experiments in disturbed sites or using artificial communities would thus not be comparable to observational drought studies.
2. In the case of observational studies, the drought year or a multi-year drought was clearly specified by the authors (that is, we did not arbitrarily extract dry years from a long-term dataset). Please note that some observational data points are from control plots of experiments (of any kind), where the authors reported that a drought had occurred during the study period. We did not involve gradient studies that compare sites of different climates, which are sometimes referred to as ‘observational studies’.
3. The paper reported the amount or proportion of change in annual or growing-season precipitation (GSP) compared with control conditions. We consistently use the term ‘control’ for normal precipitation (non-drought) year or years in observational studies and for ambient precipitation (no treatment) in experimental studies hereafter. Similarly, we use the term ‘drought’ for both drought year or years in observational studies and drought treatment in experimental studies. In the case of multi-factor experiments, where precipitation reduction was combined with any other treatment (for example, warming), data from the plots receiving drought only and data from the control plots were used.
4. The paper contained raw data on plant production under both control and drought conditions, expressed in any of the following variables: ANPP, aboveground plant biomass (in grassland studies only) or percentage plant cover. In 79% of the studies that used ANPP as a production variable, ANPP was estimated by harvesting peak or end-of-season AGB. We therefore did not distinguish between ANPP and AGB, which are referred to as ‘biomass’ hereafter. We included the papers that reported the production of the whole plant community, or at least that of the dominant species or functional groups approximating the abundance of the whole community.
5. When multiple papers were published on the same experiment or natural drought event at the same study site, the most long-term study including the largest number of drought years was chosen.

In addition to the systematic literature search, we included 27 studies (9 experimental, 17 observational and 1 that included both types) meeting the above criteria from the cited references of the Web of Science records selected for our meta-analyses, and from previous meta-analyses and reviews on the topic. In total, this resulted in 114 studies (52 experimental, 60 observational and 2 that included both types; Supplementary Note 9, Supplementary Fig. 2 and ref. ^25^).

### Data compilation

Data were extracted from the text or tables, or were read from the figures using Web Plot Digitizer^26^. For each study, we collected the study site, latitude, longitude, mean annual temperature (MAT) and precipitation (MAP), study type (experimental or observational), and drought length (the number of consecutive drought years). When MAT or MAP was not documented in the paper, it was extracted from another published study conducted at the same study site (identified by site names and geographic coordinates) or from an online climate database cited in the respective paper. We also collected vegetation type—that is, grassland when it was dominated by grasses, or shrubland when the dominant species included one or more shrub species (involving communities co-dominated by grasses and shrubs). Data from the same study (that is, paper) but from different geographic locations or environmental conditions (for example, soil types, land uses or multiple levels of experimental drought) were collected as distinct data points (but see ‘Statistical analysis’ for how these points were handled). As a result, the 114 published papers provided 239 data points (112 experimental and 127 observational)^25^.

For the observational studies, normal precipitation year or years specified by the authors was used as the control. If it was not specified in the paper, the year immediately preceding the drought year(s) was chosen as the control. When no data from the pre-drought year were available, the year immediately following the drought year(s) (14 data points) or a multi-year period given in the paper (22 data points) was used as the control. For the experimental studies, we also collected treatment size (that is, rainout shelter area or, if it was not reported in the paper, the experimental plot size).

For the calculation of drought severity, we used yearly precipitation (YP), which was reported in a much higher number of studies than GSP. We extracted YP for both control (YP_control_) and drought (YP_drought_). For the observational studies, when a multi-year period was used as the control or the natural drought lasted for more than one year, precipitation values were averaged across the control or drought years, respectively. Consistently, in the case of multi-year drought experiments, YP_control_ and YP_drought_ were averaged across the treatment years. When only GSP was published in the paper (63 of 239 data points), we used this to obtain YP data as follows: we regarded MAP as YP_control_, and YP_drought_ was calculated as YP_drought_ = MAP − (GSP_control_ − GSP_drought_). From YP_control_ and YP_drought_ data, we calculated drought severity as follows: (YP_drought_ − YP_control_)/YP_control_ × 100.

For production, we compiled the mean, replication (*N*) and, if the study reported it, a variance estimate (s.d., s.e.m. or 95% CI) for both control and drought. In the case of multi-year droughts, data only from the last drought year were extracted, except in five studies (17 data points) where production data were given as an average for the drought years. When both biomass and cover data were presented in the paper, we chose biomass. For each study, we consistently considered replication as the number of the smallest independent study unit. When only the range of replications was reported in a study, we chose the smallest number.

To quantify climatic aridity for each study site, we used an aridity index (AI), calculated as the ratio of MAP and mean annual PET (AI = MAP/PET). This is a frequently used index in recent climate change research^27,28^. AI values were extracted from the Global Aridity Index and Potential Evapotranspiration (ET0) Climate Database v.2 for the period of 1970–2000 (aggregated on annual basis)^29^.

Because we wanted to prevent our analysis from being distorted by a strongly unequal distribution of studies between the two study types regarding some potentially important explanatory variables, we left out studies from our focal meta-analysis in three steps. First, we left out studies that were conducted at wet sites—that is, where site AI exceeded 1. The value of 1 was chosen for two reasons: above this value, the distribution of studies between the two study types was extremely uneven (22 experimental versus 2 observational data points with AI > 1)^25^, and the AI value of 1 is a bioclimatically meaningful threshold, where MAP equals PET. Second, we left out shrublands, because we had only 14 shrubland studies (out of 105 studies with AI < 1), and more importantly, only 4 of these were experimental. Finally, we left out 15 grassland studies that analysed percentage cover as the biomass proxy (instead of biomass), because 12 studies (24 data points) were observational, but only 3 (4 data points) were experimental. We thus ended up with 80 studies (39 experimental, 39 observational and 2 that included both types) and 159 data points (75 experimental and 84 observational). Please note that we used only 158 data points in our focal meta-analysis (see below).

### Effect size and weighting factors

For effect size, we used lnRR, which is the most commonly used effect size metric in ecology and evolution^30^. It was calculated as ln(*D*/*C*), where *C* and *D* are the control and drought mean of production, respectively. In most meta-analyses, effect sizes are weighted by study precision, most commonly by the inverse of study variance^31^. However, the variance estimate (s.e.m., s.d. or 95% CI) was not reported by the authors in 25% of the data points of the focal dataset. In addition, the variance-based weighting function could assign extreme weights to individual studies, resulting in the average effect size being primarily determined by a small number of studies^32^. As an alternative weighting function, replication is frequently adopted in meta-analyses^33,34^. We therefore weighted lnRR by replication in our focal meta-analysis. The weight associated with each lnRR value (*W*_*i*_) was calculated as *W*_*i*_ = *N*_*i*_/∑*N*_*i*_, and *N*_*i*_ = *N*_C_ × *N*_D_/(*N*_C_ + *N*_D_), where *N*_C_ and *N*_D_ are the replication for control and drought, respectively^35^. Our focal meta-analysis included 158 data points, because the replication number (*N*) was not available for one data point of the focal dataset.

In addition to this focal replication-weighted (or *N*-weighted) meta-analysis, we conducted three meta-analyses to assess the robustness of our results. We performed (1) an unweighted meta-analysis for the focal dataset (159 data points), (2) a variance-weighted meta-analysis for a subset of our focal dataset where variance estimates were available (120 data points) and (3) a separate *N*-weighted meta-analysis for data that were left out from the focal dataset—that is, shrublands, grasslands with cover estimates and/or site AI exceeding 1 (80 data points). For the variance-weighted meta-analysis, the weights were calculated as the inverse of the pooled variance following ref. ^35^. For the experimental studies in the focal dataset (75 data points), we performed an *N*-weighted meta-analysis to test the effect of treatment size on lnRR.

### Statistical analysis

Each statistical analysis was performed in the R programming environment (v.4.1.0)^36^.

We applied meta-analytic mixed-effects models to evaluate the effects of study type and three potential confounding factors (site aridity, drought length and drought severity) on lnRR (metafor package^37^). The three continuous variables were centred to avoid multicollinearity and to get easily interpretable parameter estimates^38^. For the full models on the focal dataset, we evaluated both the main effects of the predictors and their first-order interactions with study type. For the separate *N*-weighted meta-analysis on data that were left out from the focal dataset, we tested the main effect of study type only. In the *N*-weighted meta-analysis on the experimental studies of the focal dataset, we included treatment size as a single fixed effect. Data points from the same study received a common study ID, and study ID was treated as a random effect in all models to account for the non-independence of individual effect sizes calculated from the same study. Besides the full model in each meta-analysis, we made an information-theoretic model selection based on the Akaike information criterion corrected for small sample size by using the dredge function of the MuMIn package^39^ to identify the minimum adequate model that was best supported by the data^40^. In each of the above analyses, the test assumptions were checked by visual examinations of residual diagnostic plots according to ref. ^41^, and we used DHARMa package functions for testing overdispersion and homogeneity of residual variances^42^. The presence of multicollinearity among the explanatory variables was checked with variance inflation factors. Variance inflation factors were below 3 for each term in each model (except for a single interaction term (3.11); Supplementary Note 2), suggesting that no collinearity between predictors occurred.

For each meta-analytic model, we fitted an equivalent linear mixed-effects model using the nlme package^43^, setting the residual error to 1. We used the inverse of replication and the pooled variance as weights in the *N*-weighted and variance-weighted models, respectively. In this way, we could extract analysis of variance tables showing the significance test of each fixed-effect term, and we computed *R*^2^ values as a measure of model fit according to ref. ^44^ using the r2glmm package^45^.

For the focal dataset, we tested whether experimental and observational studies differed in average site aridity, drought length, drought severity and AGB. For site aridity, we applied a beta regression with a logit link function, using the glmmTMB package^46^. The difference in drought length between experimental and observational studies was tested with a generalized mixed-effects model with a Poisson distribution and a log link function (lme4 package^47^). Linear mixed-effects models were used to assess the difference in drought severity and in AGB between the two study types (nlme package^43^). For the comparison of AGB, we used the control mean of each data point and converted the different units of biomass reported in the papers into g m^−2^. In each analysis, we used study ID as a random effect.

In addition, we considered two other potential confounding factors: plant species richness, which often positively affects primary productivity, and dominant life form (annual versus perennial), because annual-dominated ecosystems may be less resistant to drought than those dominated by herbaceous perennials^48^. However, we found very limited species richness data; it was included in only 16 studies (20% of studies). Furthermore, these data were estimated at various spatial scales (ranging from 0.04 to 10,000 m^2^) depending on the study. We therefore could not include species richness in the analysis as a potential confounding factor or even reliably compare this variable between the two study types in a separate analysis. Regarding dominant life form, the overriding dominance of perennial grasslands in our focal dataset (70 of the 80 studies) did not allow us to include this variable in our analysis.

We assessed whether publication bias could be detected for the data included in the focal meta-analysis, and for experimental and observational studies separately, by using two frequently used methods. First, we performed a file-drawer analysis with the Rosenberg method^49^ by calculating the number of studies averaging null results that would have to be added to our set of observed outcomes to reduce the combined *P* value to 0.05. Second, we assessed asymmetry in funnel plots on the basis of Egger’s regression test^50^. Both analyses were performed using the metafor package^37^.

### Reporting Summary

Further information on research design is available in the Nature Research Reporting Summary linked to this article.

## Supplementary information

Supplementary Information Supplementary Notes 1–9 and Figs. 1 and 2.

Reporting Summary

Peer Review Information

## Data Availability

The data that support the findings of this study are available in figshare^25^ with the identifier 10.6084/m9.figshare.17881073. The AI data were extracted from Global Aridity Index and Potential Evapotranspiration (ET0) Climate Database v.2, which is available in figshare^29^ with the identifier 10.6084/m9.figshare.7504448.v3.

## Code availability

The computer code (R scripts) of the analyses is available in figshare^25^ with the identifier 10.6084/m9.figshare.17881073.
