# Publisher Correction: Field experiments underestimate aboveground biomass response to drought

**DOI:** 10.1038/s41559-022-01767-2

**Published:** 2022-04-19

**Authors:** György Kröel-Dulay, Andrea Mojzes, Katalin Szitár, Michael Bahn, Péter Batáry, Claus Beier, Mark Bilton, Hans J. De Boeck, Jeffrey S. Dukes, Marc Estiarte, Petr Holub, Anke Jentsch, Inger Kappel Schmidt, Juergen Kreyling, Sabine Reinsch, Klaus Steenberg Larsen, Marcelo Sternberg, Katja Tielbörger, Albert Tietema, Sara Vicca, Josep Peñuelas

**Affiliations:** 1 Institute of Ecology and Botany, Centre for Ecological Research, Vácrátót, Hungary; 2 ‘Lendület’ Landscape and Conservation Ecology, Institute of Ecology and Botany, Centre for Ecological Research, Vácrátót, Hungary; 3 Department of Ecology, University of Innsbruck, Innsbruck, Austria; 4 Department of Geosciences and Natural Resource Management, University of Copenhagen, Frederiksberg, Denmark; 5 Namibia University of Science and Technology, Windhoek, Namibia; 6 Plants and Ecosystems (PLECO), Department of Biology, University of Antwerp, Wilrijk, Belgium; 7 Department of Forestry and Natural Resources, Purdue University, West Lafayette, IN USA; 8 Department of Biological Sciences, Purdue University, West Lafayette, IN USA; 9 CSIC, Global Ecology Unit CREAF-CSIC-UAB, Bellaterra, Spain; 10 CREAF, Cerdanyola del Vallès, Spain; 11 Global Change Research Institute of the Czech Academy of Sciences, Brno, Czech Republic; 12 Disturbance Ecology, Bayreuth Center of Ecology and Environmental Research, University of Bayreuth, Bayreuth, Germany; 13 Experimental Plant Ecology, University of Greifswald, Greifswald, Germany; 14 UK Centre for Ecology & Hydrology, Bangor, UK; 15 School of Plant Sciences and Food Security, Faculty of Life Sciences, Tel Aviv University, Tel Aviv, Israel; 16 Plant Ecology Group, University of Tübingen, Tübingen, Germany; 17 Institute for Biodiversity and Ecosystem Dynamics (IBED), Ecosystem and Landscape Dynamics (ELD), University of Amsterdam, Amsterdam, the Netherlands

**Keywords:** Ecosystem ecology, Climate-change ecology

Correction to: *Nature Ecology & Evolution* 10.1038/s41559-022-01685-3, published online 10 March 2022.

In the version of this article initially published, there were errors in the Fig. 2a,b *y*-axis labels “Effect size (lnRR).” The axis units, originally “0, 1, 2”, should instead have read “0, –1, –2”. The errors have been corrected in the HTML and PDF versions of the article.

