# Supplementary material for: Field experiments underestimate aboveground biomass response to drought
Source: Nat Ecol Evol. 2022 Mar 10;6(5):540–5. doi: 10.1038/s41559-022-01685-3 (PMC9085612; doi:10.1038/s41559-022-01685-3)
Supplement: Supplementary file 1 — Supplementary Notes 1–9 and Figs. 1 and 2. [file 41559_2022_1685_MOESM1_ESM.pdf]

nature portfolio

https://doi.org/10.1038/s41559-022-01685-3

Supplementary information

Field experiments underestimate aboveground biomass response to drought

In the format provided by the authors and unedited

Supplementary Information

to

Field experiments underestimate aboveground biomass response to drought

György Kröel-Dulay Andrea Mojzes, Katalin Szitár, Michael Bahn, Péter Batáry, Claus Beier, Mark Bilton, Hans J. De Boeck, Jeffrey S. Dukes, Marc Estiarte, Petr Holub, Anke Jentsch[1], Inger Kappel Schmidt, Juergen Kreyling, Sabine Reinsch, Klaus Steenberg Larsen, Marcelo Sternberg, Katja Tielbörger, Albert Tietema, Sara Vicca, Josep Peñuelas

Corresponding author: György Kröel-Dulay; E-mail: kroel-dulay.gyorgy@ecolres.hu

Supplementary Note 1 │ List of synthesis studies (reviews and meta-analyses) about the effect of drought on net primary production (NPP) or aboveground biomass (AGB)

1. <u>Studies covering experimental droughts only</u>

Gao J, Zhang L, Tang Z, Wu S (2019) A synthesis of ecosystem aboveground productivity and its process variables under simulated drought stress. Journal of Ecology 107: 2519-2531.

Li W, Li X, Zhao Y, Zheng S, Bai Y (2018) Ecosystem structure, functioning and stability under climate change and grazing in grasslands: current status and future prospects. Current Opinion in Environmental Sustainability 33: 124-135.

Matos IS, Menor IO, Rifai SW, Rosado, BHP (2020) Deciphering the stability of grassland productivity in response to rainfall manipulation experiments. Global Ecology and Biogeography 29: 558-572.

Orsenigo S, Mondoni A, Rossi G, Abeli T (2014) Some like it hot and some like it cold, but not too much: plant responses to climate extremes. Plant Ecology 215: 677-688.

Song J, Wan S, Piao S, Knapp AK, Classen AT, Vicca S, ..., Zheng M (2019) A meta-analysis of 1,119 manipulative experiments on terrestrial carbon-cycling responses to global change. Nature Ecology & Evolution 3: 1309-1320.

Unger S, Jongen M (2015) Consequences of changing precipitation patterns for ecosystem functioning in grasslands: a review. In: Lüttge U, Beyschlag W (eds) Progress in Botany 76. Springer, Cham, pp. 347-393.

Wang C, Sun Y, Chen HYH, Yang J, Ruan H (2021) Meta-analysis shows non-uniform responses of above-and belowground productivity to drought. Science of the Total Environment 782: 146901.

Wilcox KR, Shi Z, Gherardi LA, Lemoine NP, Koerner SE, Hoover DL, ..., Luo Y (2017) Asymmetric responses of primary productivity to precipitation extremes: a synthesis of grassland precipitation manipulation experiments. Global Change Biology 23: 4376-4385.

Wu Z, Dijkstra P, Koch GW, Peñuelas J, Hungate BA (2011) Responses of terrestrial ecosystems to temperature and precipitation change: a meta- analysis of experimental manipulation. Global Change Biology 17: 927-942.

Zhang F, Quan Q, Ma F, Tian D, Hoover DL, Zhou Q, Niu S (2019) When does extreme drought elicit extreme ecological responses? Journal of Ecology 107: 2553-2563.

Zhang C, Xi N (2021) Precipitation changes regulate plant and soil microbial biomass via plasticity in plant biomass allocation in grasslands: a meta-analysis. Frontiers in Plant Science 12: 614968.

2. <u>Studies covering observational droughts only</u>

Ruppert JC, Harmoney K, Henkin Z, Snyman HA, Sternberg M, Willms W, Linstädter A (2015) Quantifying drylands' drought resistance and recovery: the importance of drought intensity, dominant life history and grazing regime. Global Change Biology 21: 1258-1270.

3. <u>Studies covering (but not comparing) observational and experimental droughts</u>

Stuart-Haëntjens E, De Boeck HJ, Lemoine NP, Mänd P, Kröel-Dulay, Gy, Schmidt IK, Jentsch A, Stampfli A, Anderegg WRL, Bahn M, Kreyling J, Wohlgemuth T, Lloret F, Classen AT, Gough CM, Smith MD (2018) Mean annual precipitation predicts primary production resistance and resilience to extreme drought. Science of the Total Environment 636: 360-366.

**Supplementary Note 2 | ** Results of N-weighted (replication-weighted) meta-analysis with log response ratio (lnRR) on the focal data set (grassland data with biomass estimates and site aridity index below 1)

Table 1 | Parameter estimates of the final minimum adequate model with the lowest AICc. AICc: 228.4, AIC: 232.0, R-squared: 0.091, number of data points: 158. Residual heterogeneity (Q_E) is 126.9 (DF = 154, P = 0.9458).

	Coefficient	SE	z-value	P-value	CI lower	CI upper
Intercept	-0.2768	0.0841	-3.29	0.0010	-0.4416	-0.1120
Study type Observational	-0.3120	0.1102	-2.83	0.0046	-0.5280	-0.0961
Site aridity	0.5772	0.2588	2.23	0.0257	0.0700	1.0843
Drought severity	0.5969	0.3059	1.95	0.0510	-0.0027	1.1965

Table 2 | ANOVA table of the final minimum adequate model with the lowest AICc. Variance inflation factor (VIF) is shown for the fixed effects to reveal potential multicollinearity.

	numDF, denDF	F-value	P-value	VIF
Study type	1,75	6.68	0.0117	1.02
Drought severity	1,75	5.87	0.0570	1.02
Site aridity	1,75	3.74	0.0178	1.01

Table 3 | Parameter estimates of the full model including all main effects, and the interactive effects of study type and the other explanatory variables (drought severity, site aridity, and drought length). AICc: 237.9, AIC: 236.6, R-squared: 0.110, number of data points: 158. Residual heterogeneity (Q_E) is 116.8 (DF = 150, P = 0.9793).

	Coefficient	SE	z-value	P-value	CI lower	CI upper
Intercept	-0.2881	0.0784	-3.67	0.0002	-0.4418	-0.1343
Study type Observational	-0.3296	0.1017	-3.24	0.0012	-0.5290	-0.1302
Drought severity	0.4423	0.4342	1.02	0.3083	-0.4086	1.2933
Site aridity	0.2054	0.4034	0.51	0.6106	-0.5852	0.9960
Drought length	0.0293	0.0393	0.74	0.4564	-0.0478	0.1063
Study type Obs: Drought severity	0.2592	0.5890	0.44	0.6598	-0.8952	1.4137
Study type Obs: Site aridity	0.4336	0.5099	0.85	0.3951	-0.5659	1.4331
Study type Obs: Drought length	-0.0770	0.0534	-1.44	0.1491	-0.1817	0.0276

Table 4 | ANOVA table of the full model including all main effects, and the interactive effects of study type and the other explanatory variables (drought severity, site aridity, and drought length). Variance inflation factor (VIF) is shown for the fixed effects to reveal potential multicollinearity.

	numDF, denDF	F-value	P-value	VIF
Study type	1,79	87.29	0.0070	1.05
Drought severity	1,71	3.83	0.0542	2.29
Site aridity	1,71	6.72	0.0116	2.96
Drought length	1,71	0.19	0.6671	2.04
Study type: Drought severity	1,71	0.18	0.6693	2.31
Study type: Site aridity	1,71	1.28	0.2621	3.11
Study type: Drought length	1,71	1.91	0.1714	2.52

**Supplementary Note 3 | ** Results of variance-weighted meta-analysis with lnRR on a subset of the focal data set where variance estimates were available

Table 1 | Parameter estimates of the final minimum adequate model with the lowest AICc. AICc: 82.1, AIC: 81.8, R-squared: 0.154, number of data points: 120. Residual heterogeneity (Q_E) is 543.4 (DF = 116, P < 0.0001).

	Coefficient	SE	z-value	P-value	CI lower	CI upper
Intercept	-0.2679	0.0568	-4.71	<0.0001	-0.3793	-0.1565
Study type Observational	-0.2069	0.0862	-2.40	0.0163	-0.3758	-0.0381
Site aridity	0.3875	0.1484	2.61	0.0090	0.0966	0.6783
Drought severity	0.8205	0.1751	4.68	<0.0001	0.4772	1.1638

Table 2 | ANOVA table of the final minimum adequate model with the lowest AICc. Variance inflation factor (VIF) is shown for the fixed effects to reveal potential multicollinearity.

	numDF, denDF	F-value	P-value	VIF
Study type	1,60	3.54	0.0647	1.03
Drought severity	1,56	21.74	<0.0001	1.03
Site aridity	1,56	8.68	0.0047	1.01

Table 3 | Parameter estimates of the full model including all main effects, and the interactive effects of study type and the other explanatory variables (drought severity, site aridity, and drought length). AICc: 89.1, AIC: 87.4, R-squared: 0.183, number of data points: 120. Residual heterogeneity (Q_E) is 501.7 (DF = 112, P < 0.0001).

	Coefficient	SE	z-value	P-value	CI lower	CI upper
Intercept	-0.2627	0.0553	-4.75	<0.0001	-0.3711	-0.1542
Study type Observational	-0.2209	0.0871	-2.54	0.0112	-0.3915	-0.0502
Drought severity	0.7258	0.1885	3.85	0.0001	0.3563	1.0953
Site aridity	0.3665	0.1795	2.04	0.0411	0.0148	0.7182
Drought length	-0.0143	0.0221	-0.65	0.5175	-0.0577	0.0290
Study type Obs: Drought severity	0.5438	0.4879	1.12	0.2650	-0.4124	1.5000
Study type Obs: Site aridity	-0.0355	0.3234	-0.11	0.9126	-0.6693	0.5984
Study type Obs: Drought length	0.0269	0.0388	0.70	0.4877	-0.0492	0.1031

Table 4 | ANOVA table of the full model including all main effects, and the interactive effects of study type and the other explanatory variables (drought severity, site aridity, and drought length). Variance inflation factor (VIF) is shown for the fixed effects to reveal potential multicollinearity.

	numDF, denDF	F-value	P-value	VIF
Study type	1,59	3.56	0.0642	1.16
Drought severity	1,59	21.82	<0.0001	1.24
Site aridity	1,53	8.67	0.0048	1.55
Drought length	1,53	0.001	0.9749	1.67
Study type: Drought severity	1,53	1.51	0.2245	1.50
Study type: Site aridity	1,53	0.09	0.7717	1.74
Study type: Drought length	1,59	0.45	0.5048	1.87

**Supplementary Note 4 | ** Results of unweighted meta-analysis with lnRR on the focal data set (grassland data with biomass estimates and site aridity index below 1)

Table 1 | Parameter estimates of the final minimum adequate model with the lowest AICc. AICc: 327.0, AIC: 330.5, R-squared: 0.017, number of data points: 159. Residual heterogeneity (Q_E) is 32.3 (DF = 157, P = 1).

	Coefficient	SE	z-value	P-value	CI lower	CI upper
Intercept	-0.3249	0.1155	-2.81	0.0049	-0.5513	-0.0986
Study type Observational	-0.2644	0.1589	-1.66	0.0961	-0.5757	0.0470

Table 2 | ANOVA table of the final minimum adequate model with the lowest AICc.

	numDF, denDF	F-value	P-value
Study type	1,78	2.77	0.1001

Table 3 | Parameter estimates of the full model including all main effects, and the interactive effects of study type and the other explanatory variables (drought severity, site aridity, and drought length). AICc: 340.6, AIC: 339.4, R-squared: 0.036, number of data points: 159. Residual heterogeneity (Q_E) is 29.2 (DF = 151, P = 1).

	Coefficient	SE	z-value	P-value	CI lower	CI upper
Intercept	-0.3187	0.1188	-2.68	0.0073	-0.5515	-0.0858
Study type Observational	-0.2869	0.1641	-1.75	0.0804	-0.6085	0.0347
Drought severity	0.4387	0.7136	0.61	0.5387	-0.9599	1.8372
Site aridity	0.2725	0.5986	0.46	0.6490	-0.9007	1.4457
Drought length	0.0166	0.0503	0.03	0.7419	-0.0821	0.1153
Study type Obs: Drought severity	0.0608	1.0453	0.06	0.9536	-1.9879	2.1095
Study type Obs: Site aridity	0.3993	0.8249	0.48	0.6284	-1.2175	2.0161
Study type Obs: Drought length	-0.0181	0.0782	-0.23	0.8165	-0.1714	0.1351

Table 4 | ANOVA table of the full model including all main effects, and the interactive effects of study type and the other explanatory variables (drought severity, site aridity, and drought length). Variance inflation factor (VIF) is shown for the fixed effects to reveal potential multicollinearity.

	numDF, denDF	F-value	P-value	VIF
Study type	1,79	2.77	0.1004	1.07
Drought severity	1,72	0.91	0.3434	1.99
Site aridity	1,72	1.81	0.1827	2.31
Drought length	1,72	0.03	0.8575	1.88
Study type: Drought severity	1,72	0.01	0.9332	2.05
Study type: Site aridity	1,72	0.30	0.5875	2.48
Study type: Drought length	1,72	0.05	0.8172	2.00

**Supplementary Note 5 | ** Results of the separate N-weighted meta-analysis with lnRR on the data that were left out from the focal data set (shrublands, grasslands with cover estimates and/or site aridity index exceeding 1)

Table 1 | Parameter estimates of the model with study type as a single explanatory variable. AICc: 111.2, AIC: 110.9, R-squared: 0.04, number of data points: 80. Residual heterogeneity (Q_E) is 39.6 (DF = 78, P = 0.9999).

	Coefficient	SE	z-value	P-value	CI lower	CI upper
Intercept	-0.2532	0.1090	-2.32	0.0202	-0.4670	-0.0395
Study type Observational	-0.2716	0.1274	-2.13	0.0330	-0.5214	-0.0219

Table 2 | ANOVA table of the model with study type as a single explanatory variable.

	numDF, denDF	F-value	P-value
Study type	1,34	4.43	0.0427

Supplementary Note 6 | Results of the comparison of site aridity, drought length, drought severity, and aboveground biomass (biomass data for non-drought year(s) in observational studies and for control plots in experimental studies) between the two study types of the focal data set (grassland data with biomass estimates and site aridity index below 1)

Table 1 | Mean and standard error of the mean (SE) of site aridity, drought length, drought severity, and aboveground biomass for the two study types (number of data points: 75 experimental and 84 observational).

	Mean		SE	
Study type	Experimental	Observational	Experimental	Observational
Site aridity	0.356	0.369	0.0225	0.0229
Drought length (years)	2.750	1.950	0.2670	0.2150
Drought severity	-0.386	-0.337	0.0188	0.0163
Biomass (g m^{-2})	249.1	257.4	34.32	18.57

Table 2 | Parameter estimates of the beta regression model used for the comparison of site aridity between the two study types with logit link function.

	Coefficient	SE	z-value	P-value
Intercept	-0.5322	0.1349	-3.95	<0.0001
Study type Obs	-0.1788	0.1745	-1.02	0.3060

Table 3 | Parameter estimates of the generalized mixed-effects model used for the comparison of drought length between the two study types with Poisson distribution and log link function.

	Coefficient	SE	z-value	P-value
Intercept	0.8665	0.1238	7.00	<0.0001
Study type Obs	-0.3828	0.1743	-2.20	0.0280

Table 4 | Parameter estimates of the linear mixed-effects model used for the comparison of drought severity between the two study types.

	Coefficient	SE	DF	t-value	P-value
Intercept	-0.3824	0.0239	79	-15.98	<0.0001
Study type Obs	0.0271	0.0322	78	0.84	0.4031

Table 5 | Parameter estimates of the linear mixed-effects model used for the comparison of aboveground biomass between the two study types. Biomass data were log-transformed to fulfil model assumptions.

	Coefficient	SE	DF	t-value	P-value
Intercept	5.1811	0.1204	79	43.03	<0.0001
Study type Obs	0.0927	0.1633	78	0.57	0.5718

Supplementary Note 7 | Results of the tests for detecting publication bias on the focal data set (grassland data with biomass estimates and site aridity index below 1)

Table 1 | Results of the fail-safe N calculation using the Rosenberg method.

	Experimental	Observational	Whole focal data set
Fail-safe N	373	3202	6472
Significance level	< 0.0001	< 0.0001	< 0.0001

Table 2 | Results of the Egger's regression test for funnel plot asymmetry based on N-weighted (replication-weighted) meta-analytic model with log response ratio (lnRR).

	Experimental	Observational	Whole focal data set
z-value	-0.1474	0.3173	1.1383
P-value	0.8828	0.7510	0.2550

Fig. 1 | Funnel plot of N-weighted meta-analytic model with lnRR (focal meta-analysis) on the experimental studies (number of data points: 75).

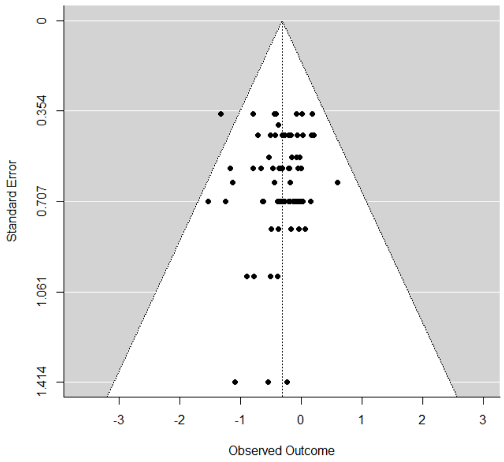

Fig. 2 | Funnel plot of N-weighted meta-analytic model with lnRR (focal meta-analysis) on the observational studies (number of data points: 83).

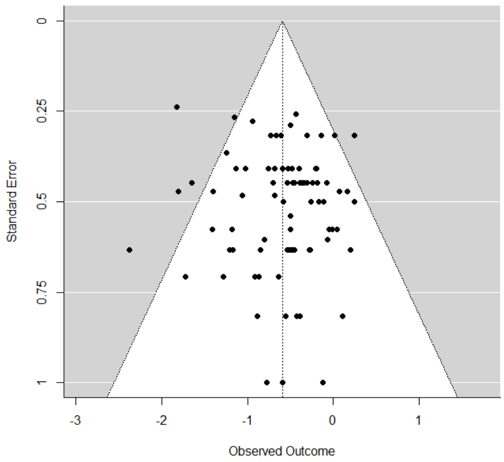

Fig. 3 | Funnel plot of N-weighted meta-analytic model with lnRR (focal meta-analysis) on the whole focal data set (number of data points: 158).

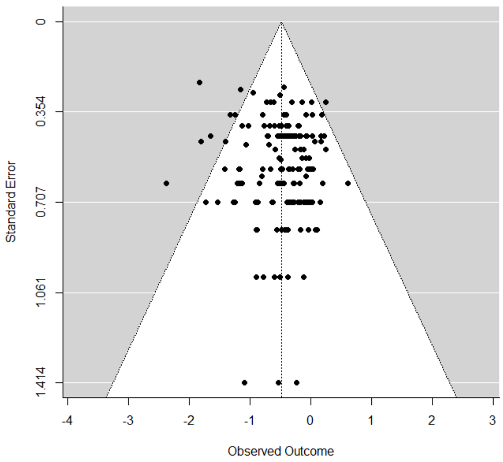

Supplementary Note 8 | Results of N-weighted (replication-weighted) meta-analysis with lnRR on the experimental data points of the focal data set (grassland data with biomass estimates and site aridity index below 1) with treatment size (i.e. rainout shelter area, or if it was not reported in the paper, the experimental plot size) as a single explanatory variable

Table 1 | Parameter estimates of the model (number of data points: 75). Residual heterogeneity (Q_E) is 33.1 (DF = 73, P = 1).

	Coefficient	SE	z-value	P-value	CI lower	CI upper
Intercept	-0.3316	0.0673	-4.93	<0.0001	-0.4636	-0.1997
Treatment size	-0.0008	0.0007	-1.17	0.2415	-0.0021	0.0005

Table 2 | ANOVA table of the model.

	numDF, denDF	F-value	P-value
Treatment size	1,33	1.34	0.2562

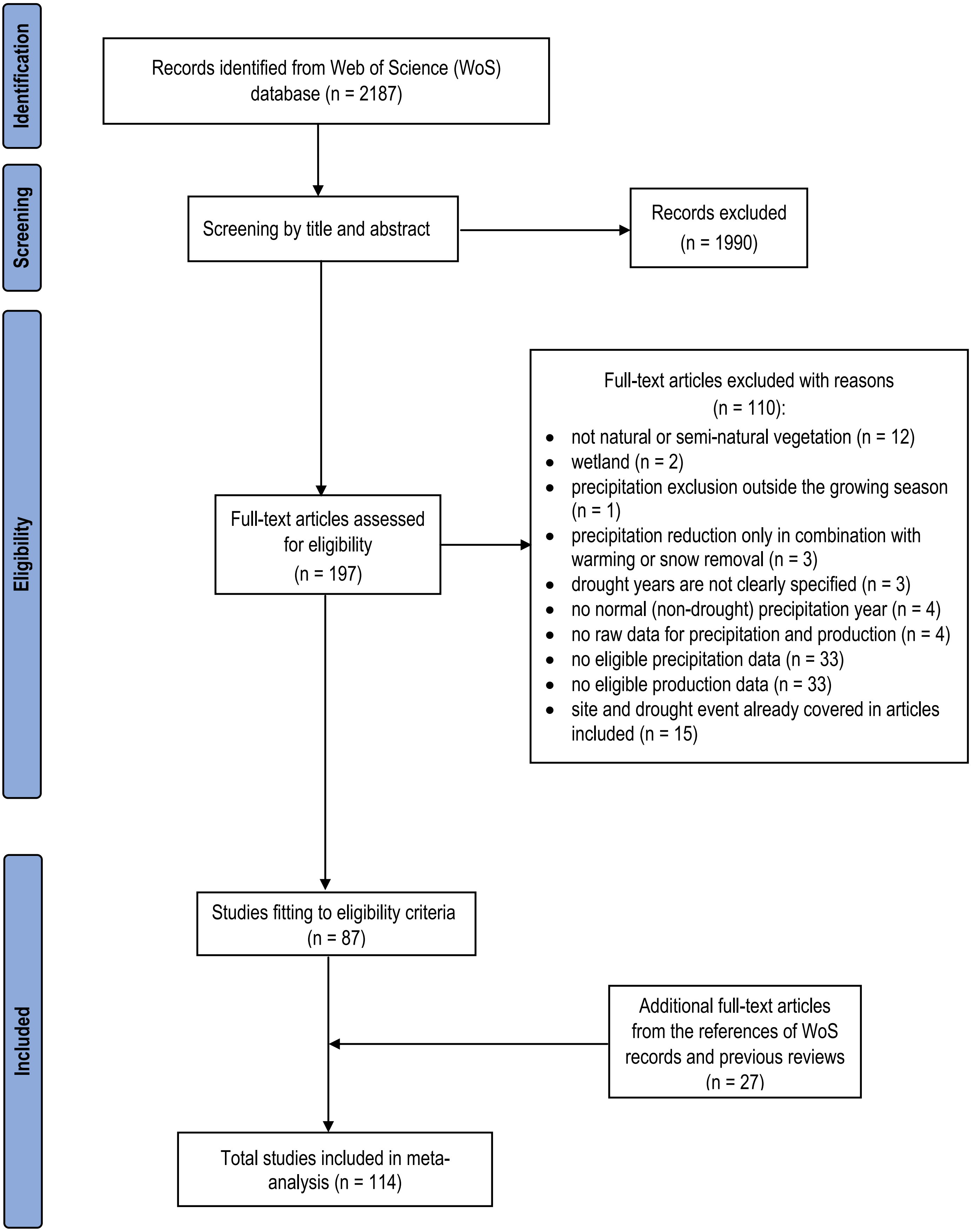

Supplementary Figure 1 | PRISMA flow diagram describing the steps of selecting articles for inclusion in the meta-analysis

**Supplementary Note 9 | ** List of studies found in the literature search. The number of the paper corresponds to the study ID in the table of collected data (available in Figshare: https://doi.org/10.6084/m9.figshare.17881073). Shaded studies at the end of the list were not included in the focal meta-analysis, but were included in a separate replication-weighted meta-analysis (see Methods for details)

1. Abrams MD, Knapp AK, Hulbert LC (1986) A ten- year record of aboveground biomass in a Kansas tallgrass prairie: effects of fire and topographic position. American Journal of Botany 73: 1509-1515.
2. Aires LMI, Pio CA, Pereira JS (2008) Carbon dioxide exchange above a Mediterranean C3/C4 grassland during two climatologically contrasting years. Global Change Biology 14: 539-555.
3. Alon M, Sternberg M (2019) Effects of extreme drought on primary production, species composition and species diversity of a Mediterranean annual plant community. Journal of Vegetation Science 30: 1045-1055.
4. Arredondo T, Garcìa-Moya E, Huber-Sannwald E, Loescher HW, Delgado-Balbuena J, Luna-Luna M (2016) Drought manipulation and its direct and legacy effects on productivity of a monodominant and mixed-species semi-arid grassland. Agricultural and Forest Meteorology 223: 132-140.
5. Baoyin T, Li FY, Bao Q, Minggagud H, Zhong Y (2014) Effects of mowing regimes and climate variability on hay production of *Leymus chinensis* (Trin.) Tzvelev grassland in northern China. The Rangeland Journal 36: 593-600.
6. Bat-Oyun T, Shinoda M, Cheng Y, Purevdorj Y (2016) Effects of grazing and precipitation variability on vegetation dynamics in a Mongolian dry steppe. Journal of Plant Ecology 9: 508-519.
7. Briggs JM, Knapp AK (1995) Interannual variability in primary production in tallgrass prairie: climate, soil moisture, topographic position, and fire as determinants of aboveground biomass. American Journal of Botany 82: 1024-1030.
8. Brown JR, Archer S (1999) Shrub invasion of grassland: recruitment is continuous and not regulated by herbaceous biomass or density. Ecology 80: 2385-2396.
9. Byrne KM, Lauenroth WK, Adler PB (2013) Contrasting effects of precipitation manipulations on production in two sites within the central grassland region, USA. Ecosystems 16: 1039-1051.
10. Canarini A, Mariotte P, Ingram L, Merchant A, Dijkstra FA (2018) Mineral-associated soil carbon is resistant to drought but sensitive to legumes and microbial biomass in an Australian grassland. Ecosystems 21: 349-359.
11. Chen J, Shao C, Jiang S, Qu L, Zhao F, Dong G (2019) Effects of changes in precipitation on energy and water balance in a Eurasian meadow steppe. Ecological Processes 8: 17.
12. Cherwin K, Knapp A (2012) Unexpected patterns of sensitivity to drought in three semi-arid grasslands. Oecologia 169: 845-852.
13. Chieppa J, Nielsen UN, Tissue DT, Power SA (2019) Drought and phosphorus affect productivity of a mesic grassland via shifts in root traits of dominant species. Plant and Soil 444: 457-473.
14. Chimner RA, Welker JM, Morgan J, LeCain D, Reeder J (2010) Experimental manipulations of winter snow and summer rain influence ecosystem carbon cycling in a mixed-grass prairie, Wyoming, USA. Ecohydrology 3: 284-293.
15. Coupe MD, Stacey JN, Cahill Jr JF (2009) Limited effects of above- and belowground insects on community structure and function in a species- rich grassland. Journal of Vegetation Science 20: 121-129.

16. Czóbel Sz, Szirmai O, Németh Z, Gyuricza Cs, Házi J, Tóth A, Schellenberger J, Vasa L, Penksza K (2012) Short-term effects of grazing exclusion on net ecosystem CO_2 exchange and net primary production in a Pannonian sandy grassland. Notulae Botanicae Horti Agrobotanici Cluj-Napoca 40: 67-72.
17. Denton EM, Dietrich JD, Smith MD, Knapp AK (2017). Drought timing differentially affects above-and belowground productivity in a mesic grassland. Plant Ecology 218: 317-328.
18. Dong G, Guo J, Chen J, Sun G, Gao S, Hu L, Wang Y (2011) Effects of spring drought on carbon sequestration, evapotranspiration and water use efficiency in the Songnen meadow steppe in northeast China. Ecohydrology 4: 211-224.
19. Erichsen-Arychuk C, Bork EW, Bailey AW (2002) Northern dry mixed prairie responses to summer wildlife and drought. Journal of Range Management 164-170.
20. Evans SE, Burke IC (2013) Carbon and nitrogen decoupling under an 11-year drought in the shortgrass steppe. Ecosystems 16: 20-33.
21. Fahnestock JT, Detling JK (1999) Plant responses to defoliation and resource supplementation in the Pryor Mountains. Journal of Range Management 263-270.
22. Fay PA, Blair JM, Smith MD, Nippert JB, Carlisle JD, Knapp AK (2011) Relative effects of precipitation variability and warming on tallgrass prairie ecosystem function. Biogeosciences 8: 3053-3068.
23. February EC, Higgins SI, Bond WJ, Swemmer L (2013) Influence of competition and rainfall manipulation on the growth responses of savanna trees and grasses. Ecology 94: 1155-1164.
24. Fiala K, Tůma I, Holub P (2011) Effect of nitrogen addition and drought on above-ground biomass of expanding tall grasses *Calamagrostis epigejos* and *Arrhenatherum elatius*. Biologia 66: 275-281.
25. Flanagan LB, Adkinson AC (2011) Interacting controls on productivity in a northern Great Plains grassland and implications for response to ENSO events. Global Change Biology 17: 3293-3311.
26. Flanagan LB, Sharp EJ, Letts MG (2013) Response of plant biomass and soil respiration to experimental warming and precipitation manipulation in a Northern Great Plains grassland. Agricultural and Forest Meteorology 173: 40-52.
27. Frank DA (2007) Drought effects on above- and belowground production of a grazed temperate grassland ecosystem. Oecologia 152: 131-139.
28. Gamoun M (2016) Rain use efficiency, primary production and rainfall relationships in desert rangelands of Tunisia. Land Degradation and Development 27: 738-747.
29. Gao Y, Giese M, Brueck H, Yang H, Li Z (2013) The relation of biomass production with leaf traits varied under different land-use and precipitation conditions in an Inner Mongolia steppe. Ecological Research 28: 1029-1043.
30. Griffin-Nolan RJ, Carroll CJW, Denton EM, Johnston MK, Collins SL, Smith MD, Knapp AK (2018) Legacy effects of a regional drought on aboveground net primary production in six central US grasslands. Plant Ecology 219: 505-515.
31. Haddad NM, Tilman D, Knops JMH (2002) Long- term oscillations in grassland productivity induced by drought. Ecology Letters 5: 110-120.
32. Haferkamp MR, Heitschmidt RK, Karl MG (1997) Influence of Japanese brome on western wheatgrass yield. Journal of Range Management 50: 44-50.
33. Harrison SP, LaForgia ML, Latimer AM (2018) Climate- driven diversity change in annual grasslands: Drought plus deluge does not equal normal. Global Change Biology 24: 1782-1792.
34. Hein L (2006) The impacts of grazing and rainfall variability on the dynamics of a Sahelian rangeland. Journal of Arid Environments 64: 488-504.

35. Heitschmidt RK, Vermeire LT (2006) Can abundant summer precipitation counter losses in herbage production caused by spring drought? Rangeland Ecology and Management 59: 392-399.
36. Heitschmidt RK, Dowhower SL, Walker JW (1987) 14- vs. 42-paddock rotational grazing: aboveground biomass dynamics, forage production, and harvest efficiency. Journal of Range Management: 216-223.
37. Heitschmidt RK, Haferkamp MR, Karl MG, Hild AL (1999) Drought and grazing: I. Effects on quantity of forage produced. Journal of Range Management 2: 440-446.
38. Heitschmidt RK, Klement KD, Haferkamp MR (2005) Interactive effects of drought and grazing on Northern Great Plains rangelands. Rangeland Ecology and Management 58: 11-19.
39. Herbel CH, Ares FN, Wright RA (1972) Drought effects on a semidesert grassland range. Ecology 53: 1084-1093.
40. Hoeppner SS, Dukes JS (2012) Interactive responses of old-field plant growth and composition to warming and precipitation. Global Change Biology 18: 1754-1768.
41. Holub P, Tůma I, Záhora J, Fiala K (2015) Biomass production of different grassland communities under artificially modified amount of rainfall. Polish Journal of Ecology 63: 320-332.
42. Hoover DL, Knapp AK, Smith MD (2014) Resistance and resilience of a grassland ecosystem to climate extremes. Ecology 95: 2646-2656.
43. Huang L, Wang D, Yao L, Li X, Wang D, Du Q, Zhang Y, Hou X, Guo Y (2019) Primary limitation on vegetation productivity shifts from precipitation in dry years to nitrogen in wet years in a degraded arid steppe of Inner Mongolia, northern China. Journal of Soils and Sediments 19: 544-556.
44. Hulett GK, Tomanek GW (1969) Forage production on a clay upland range site in western Kansas. Journal of Range Management 22: 270-276.
45. Jamiyansharav K, Ojima D, Pielke RA, Parton W, Morgan J, Beltrán-Przekurat A, LeCain D, Smith D (2011) Seasonal and interannual variability in surface energy partitioning and vegetation cover with grazing at shortgrass steppe. Journal of Arid Environments 75: 360-370.
46. Knapp AK (1984) Water relations and growth of three grasses during wet and drought years in a tallgrass prairie. Oecologia 65: 35-43.
47. Koerner SE, Collins SL (2014) Interactive effects of grazing, drought, and fire on grassland plant communities in North America and South Africa. Ecology 95: 98-109.
48. Lauenroth WK, Sala OE (1992) Long- term forage production of North American shortgrass steppe. Ecological applications 2: 397-403.
49. Lemoine NP, Smith MD (2019) Drought and small-bodied herbivores modify nutrient cycling in the semi-arid shortgrass steppe. Plant Ecology 220: 227-239.
50. Liu Y, Pan Q, Liu H, Bai Y, Simmons M, Dittert K, Han X (2011) Plant responses following grazing removal at different stocking rates in an Inner Mongolia grassland ecosystem. Plant and Soil 340: 199-213.
51. Liu H, Mi Z, Lin L, Wang Y, Zhang Z, Zhang F, Wang H, Liu L, Zhu B, Cao G, Zhao X, Sanders NJ, Classen AT, Reich PB, He JS (2018) Shifting plant species composition in response to climate change stabilizes grassland primary production. Proceedings of the National Academy of Sciences of the United States of America 115: 4051-4056.
52. López-Sánchez A, San Miguel A, Dirzo R, Roig S (2016) Scattered trees and livestock grazing as keystones organisms for sustainable use and conservation of Mediterranean dehesas. Journal for Nature Conservation 33: 58-67.
53. Lü XT, Liu ZY, Hu YY, Zhang HY (2018) Testing nitrogen and water co-limitation of primary productivity in a temperate steppe. Plant and Soil 432: 119-127.

54. Ma W, Liu Z, Wang Z, Wang W, Liang C, Tang Y, He J-S, Fang J (2010) Climate change alters interannual variation of grassland aboveground productivity: evidence from a 22-year measurement series in the Inner Mongolian grassland. Journal of Plant Research 123: 509-517.

55. Mamolos AP, Veresoglou DS, Noitsakis V, Gerakis A (2001) Differential drought tolerance of five coexisting plant species in Mediterranean lowland grasslands. Journal of Arid Environments 49: 329-341.

56. Meng B, Shi B, Zhong S, Chai H, Li S, Wang Y, Henry HAL, Ma JY, Sun W (2019) Drought sensitivity of aboveground productivity in *Leymus chinensis* meadow steppe depends on drought timing. Oecologia 191: 685-696.

57. Miao Y, Han H, Du Y, Zhang Q, Jiang L, Hui D, Wan S (2017) Nonlinear responses of soil respiration to precipitation changes in a semiarid temperate steppe. Scientific Reports 7: 45782.

58. Miranda JD, Padilla FM, Lázaro R, Pugnaire FI (2009) Do changes in rainfall patterns affect semiarid annual plant communities? Journal of Vegetation Science 20: 269-276.

59. Miranda JD, Armas C, Padilla FM, Pugnaire FI (2011) Climatic change and rainfall patterns: Effects on semi-arid plant communities of the Iberian Southeast. Journal of Arid Environments 75: 1302-1309.

60. Muldavin EH, Moore DI, Collins SL, Wetherill KR, Lightfoot DC (2008) Aboveground net primary production dynamics in a northern Chihuahuan Desert ecosystem. Oecologia 155: 123-132.

61. Munson SM, Bunting EL, Bradford JB, Butterfield BJ, Gremer JR (2019) Plant production responses to precipitation differ along an elevation gradient and are enhanced under extremes. Ecosystems 22: 699-708.

62. Otieno DO, K'Otuto GO, Maina JN, Kuzyakov Y, Onyango JC (2010) Responses of ecosystem carbon dioxide fluxes to soil moisture fluctuations in a moist Kenyan savanna. Journal of Tropical Ecology 26: 605-618.

63. Peters DPC, Yao J, Sala OE, Anderson JP (2012) Directional climate change and potential reversal of desertification in arid and semiarid ecosystems. Global Change Biology 18: 151-163.

64. Piper JK (1995) Composition of prairie plant communities on productive versus unproductive sites in wet and dry years. Canadian Journal of Botany 73: 1635-1644.

65. Potts DL, Suding KN, Winston GC, Rocha AV, Goulden ML (2012) Ecological effects of experimental drought and prescribed fire in a southern California coastal grassland. Journal of Arid Environments 81: 59-66.

66. Power SA, Barnett KL, Ochoa-Hueso R, Facey SL, Gibson-Forty EVJ, Hartley, SE, Nielsen UN, Tissue DT, Johnson SN (2016) DRI-Grass: a new experimental platform for addressing grassland ecosystem responses to future precipitation scenarios in south-east Australia. Frontiers in Plant Science 7: 1373.

67. Puritty CE, Esch EH, Castro SP, Ryan EM, Lipson DA, Cleland EE (2019) Drought in Southern California coastal sage scrub reduces herbaceous biomass of exotic species more than native species, but exotic growth recovers quickly when drought ends. Plant Ecology 220: 151-169.

68. Shinoda M, Nachinshonhor GU, Nemoto M (2010) Impact of drought on vegetation dynamics of the Mongolian steppe: A field experiment. Journal of Arid Environments 74: 63-69.

69. Su F, Wei Y, Wang F, Guo J, Zhang J, Wang Y, Guo H, Hu S (2019) Sensitivity of plant species to warming and altered precipitation dominates the community productivity in a semiarid grassland on the Loess Plateau. Ecology and Evolution, 9(13), 7628-7638.

70. Symstad AJ, Tilman D (2001) Diversity loss, recruitment limitation, and ecosystem functioning: lessons learned from a removal experiment. Oikos 92: 424-435.
71. Tielbörger K, Bilton MC, Metz J, Kigel J, Holzapfel C, Lebrija-Trejos E, Konsens I, Parag HA, Sternberg M (2014) Middle-Eastern plant communities tolerate 9 years of drought in a multi-site climate manipulation experiment. Nature Communications 5: 5102.
72. Valkó O, Török P, Matus G, Tóthmérész B (2012) Is regular mowing the most appropriate and cost-effective management maintaining diversity and biomass of target forbs in mountain hay meadows? Flora 207: 303-309.
73. VanderWeide BL, Hartnett DC (2015) Belowground bud bank response to grazing under severe, short-term drought. Oecologia 178: 795-806.
74. VanderWeide BL, Hartnett DC, Carter DL (2014) Belowground bud banks of tallgrass prairie are insensitive to multi-year, growing-season drought. Ecosphere 5: 103.
75. Wei L, Liu J, Su J, Jing G, Zhao J, Cheng J, Jin J (2016a) Effect of clipping on soil respiration components in temperate grassland of Loess Plateau. European Journal of Soil Biology 75: 157-167.
76. Wei X, Zhang Y, Liu J, Gao H, Fan J, Jia X, Cheng J, Shao M, Zhang X (2016b) Response of soil CO_2 efflux to precipitation manipulation in a semiarid grassland. Journal of Environmental Sciences 45: 207-214.
77. Xiao X, Jiang S, Wang Y, Ojima DS, Bonham CD (1996) Temporal variation in aboveground biomass of *Leymus chinense* steppe from species to community levels in the Xilin River Basin, Inner Mongolia, China. Vegetatio 123: 1-12.
78. Xu X, Sherry RA, Niu S, Li D, Luo Y (2013) Net primary productivity and rain-use efficiency as affected by warming, altered precipitation, and clipping in a mixed-grass prairie. Global Change Biology 19: 2753-2764.
79. Zhang B, Cadotte MW, Chen S, Tan X, You C, Ren T, Chen M, Wang S, Li W, Chu C, Jiang L, Bai Y, Huang J, Han X (2019a) Plants alter their vertical root distribution rather than biomass allocation in response to changing precipitation. Ecology 100: e02828.
80. Zhang H, Yu H, Zhou C, Zhao H, Qian X (2019b) Aboveground net primary productivity not CO_2 exchange remain stable under three timing of extreme drought in a semi-arid steppe. PLoS ONE 14: e0214418.
81. Albertson FW, Weaver JE (1942) History of the native vegetation of western Kansas during seven years of continuous drought. Ecological Monographs 12: 23-51.
82. Báez S, Collins SL, Pockman WT, Johnson JE, Small EE (2013) Effects of experimental rainfall manipulations on Chihuahuan Desert grassland and shrubland plant communities. Oecologia 172: 1117-1127.
83. Bock CE, Bock JH (1999) Response of winter birds to drought and short- duration grazing in southeastern Arizona. Conservation Biology 13: 1117-1123.
84. Copeland SM, Harrison SP, Latimer AM, Damschen EI, Eskelinen AM, Fernandez- Going B, Spasojevic MJ, Anacker BL, Thorne JH (2016) Ecological effects of extreme drought on Californian herbaceous plant communities. Ecological Monographs 86: 295-311.
85. Darenova E, Holub P, Krupkova L, Pavelka M (2017) Effect of repeated spring drought and summer heavy rain on managed grassland biomass production and CO_2 efflux. Journal of Plant Ecology 10: 476-485.
86. Deléglise C, Meisser M, Mosimann E, Spiegelberger T, Signarbieux C, Jeangros B, Buttler A (2015) Drought-induced shifts in plants traits, yields and nutritive value under realistic grazing and mowing managements in a mountain grassland. Agriculture, Ecosystems and Environment 213: 94-104.

87. Domínguez MT, Sowerby A, Smith AR, Robinson DA, Van Baarsel S, Mills RTE, Marshall MR, Koller E, Lebron I, Hall J, Emmett BA (2015) Sustained impact of drought on wet shrublands mediated by soil physical changes. Biogeochemistry 122: 151-163.
88. Finger R, Gilgen AK, Prechsl UE, Buchmann N (2013) An economic assessment of drought effects on three grassland systems in Switzerland. Regional Environmental Change 13: 365-374.
89. Gibbens RP, Beck RF (1988) Changes in grass basal area and forb densities over a 64-year period on grassland types of the Jornada Experimental Range. Journal of Range Management 41: 186-192.
90. Gilgen AK, Buchmann N (2009) Response of temperate grasslands at different altitudes to simulated summer drought differed but scaled with annual precipitation. Biogeosciences 6: 5217-5250.
91. Hobbs RJ, Mooney HA (1991) Effects of rainfall variability and gopher disturbance on serpentine annual grassland dynamics. Ecology 72: 59-68.
92. Hopkins B (1978) The effects of the 1976 drought on chalk grassland in Sussex, England. Biological Conservation 14: 1-12.
93. Kimball S, Principe Z, Deutschman D, Strahm S, Huxman TE, Lulow M, Balazs K (2018) Resistance and resilience: ten years of monitoring shrub and prairie communities in Orange County, CA, USA. Ecosphere 9: e02212.
94. Kröel-Dulay Gy, Ransijn J, Schmidt IK, Beier C, De Angelis P, de Dato G, Dukes JS, Emmett B, Estiarte M, Garadnai J, Kongstad J, Kovács-Láng E, Larsen KS, Liberati D, Ogaya R, Riis-Nielsen T, Smith A, Sowerby A, Tietema A, Peñuelas J (2015) Increased sensitivity to climate change in disturbed ecosystems. Nature Communications 6: 6682.
95. Kübert A, Götz M, Kuester E, Piayda A, Werner C, Rothfuss Y, Dubbert M (2019) Nitrogen loading enhances stress impact of drought on a semi-natural temperate grassland. Frontiers in Plant Science 10: 1051.
96. Ladwig LM, Collins SL, Ford PL, White LB (2014) Chihuahuan Desert grassland responds similarly to fall, spring, and summer fires during prolonged drought. Rangeland Ecology and Management 67: 621-628.
97. Maalouf JP, Le Bagousse-Pinguet Y, Marchand L, Bâchelier E, Touzard B, Michalet R (2012) Integrating climate change into calcareous grassland management. Journal of Applied Ecology 49: 795-802.
98. Milton SJ, Dean WRJ, Marincowitz CP, Kerley GIH (1995) Effects of the 1990/91 drought on rangeland in the Steytlerville Karoo. South African Journal of Science 91: 78-84.
99. Morecroft MD, Masters GJ, Brown VK, Clarke IP, Taylor ME, Whitehouse AT (2004) Changing precipitation patterns alter plant community dynamics and succession in an ex- arable grassland. Functional Ecology 18: 648-655.
100. O'Connor TG (1995) Transformation of a savanna grassland by drought and grazing. African Journal of Range and Forage Science 12: 53-60.
101. Pechanec JF, Pickford GD, Stewart G (1937) Effects of the 1934 drought on native vegetation of the upper Snake River plains, Idaho. Ecology 18: 490-505.
102. Read JL (2004) Catastrophic drought-induced die-off of perennial chenopod shrubs in arid Australia following intensive cattle browsing. Journal of Arid Environments 58: 535-544.
103. Rondeau RJ, Pearson KT, Kelso S (2013) Vegetation response in a Colorado grassland-shrub community to extreme drought: 1999-2010. The American Midland Naturalist 170: 14-25.
104. Scott RL, Hamerlynck EP, Jenerette GD, Moran MS, Barron-Gafford GA (2010) Carbon dioxide exchange in a semidesert grassland through drought-induced vegetation change. Journal of Geophysical Research 115: G03026.

105. Serafini J, Grogan P, Aarssen L (2019) Summer precipitation limits plant species richness but not overall productivity in a temperate mesic old- field meadow. Journal of Vegetation Science 30: 832-844.
106. Souther S, Loeser M, Crews TE, Sisk T (2019) Complex response of vegetation to grazing suggests need for coordinated, landscape-level approaches to grazing management. Global Ecology and Conservation 20: e00770.
107. Stampfli A (1995) Species composition and standing crop variation in an unfertilized meadow and its relationship to climatic variability during six years. Folia Geobotanica 30(2): 117-130.
108. Stampfli A, Bloor JMG, Fischer M, Zeiter M (2018) High land- use intensity exacerbates shifts in grassland vegetation composition after severe experimental drought. Global Change Biology 24: 2021-2034.
109. Tredennick AT, Kleinhesselink AR, Taylor JB, Adler PB (2018) Ecosystem functional response across precipitation extremes in a sagebrush steppe. PeerJ 6: e4485.
110. Vermeire LT, Crowder JL, Wester DB (2014) Semiarid rangeland is resilient to summer fire and postfire grazing utilization. Rangeland Ecology and Management 67: 52-60.
111. West NE, Yorks TP (2006) Long-term interactions of climate, productivity, species richness, and growth form in relictual sagebrush steppe plant communities. Western North American Naturalist 66: 502-526.
112. White CS, Loftin SR (2000) Response of 2 semiarid grasslands to cool-season prescribed fire. Journal of Range Management 53: 52-61.
113. Yahdjian L, Sala OE (2006) Vegetation structure constrains primary production response to water availability in the Patagonian steppe. Ecology 87: 952-962.
114. Zimmer HC, Mavromihalis J, Turner VB, Moxham C, Liu C (2010) Native grasslands in the *PlainsTender* incentive scheme: conservation value, management and monitoring. The Rangeland Journal 32: 205-214.

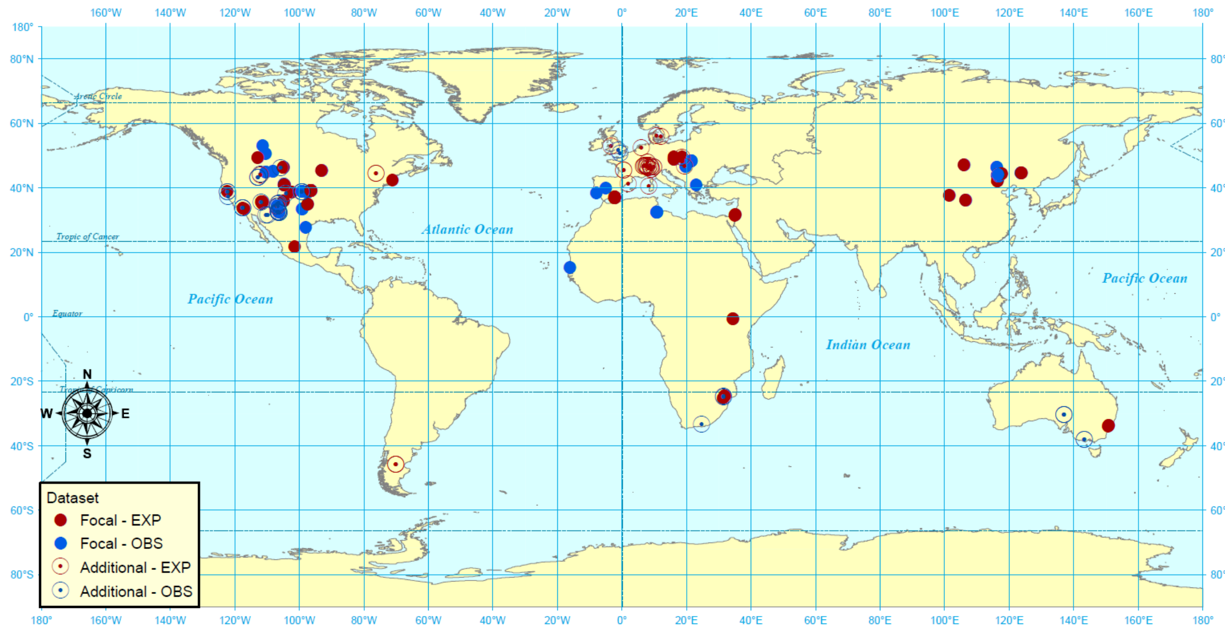

Supplementary Figure 2 | Geographic setting of the studies included in the meta-analysis. EXP stands for experimental studies, and OBS stands for observational studies. Focal-EXP and Focal-OBS studies were included in the focal analysis, while Additional-EXP and Additional-OBS studies were left out of the focal analysis for various methodological reasons, but were analysed separately (See Methods for details). This map was created using ArcGIS® software by Esri. ArcGIS® is the intellectual property of Esri and is used herein under license. Copyright © Esri. All rights reserved.
