# Supplementary material for: Field experiments underestimate aboveground biomass response to drought
Source: Nat Ecol Evol. 2022 Mar 10;6(5):540–5. doi: 10.1038/s41559-022-01685-3 (PMC9085612; doi:10.1038/s41559-022-01685-3)
Supplement: Supplementary file 2 — Reporting Summary [file 41559_2022_1685_MOESM2_ESM.pdf]

nature portfolio

Corresponding author(s): György Kröel-Dulay

Last updated by author(s): Jan 14, 2022

Reporting Summary

Nature Portfolio wishes to improve the reproducibility of the work that we publish. This form provides structure for consistency and transparency in reporting. For further information on Nature Portfolio policies, see our Editorial Policies and the Editorial Policy Checklist.

Statistics

For all statistical analyses, confirm that the following items are present in the figure legend, table legend, main text, or Methods section.

n/a	Confirmed	
☐	☒	The exact sample size (*n*) for each experimental group/condition, given as a discrete number and unit of measurement
☐	☒	A statement on whether measurements were taken from distinct samples or whether the same sample was measured repeatedly
☐	☒	The statistical test(s) used AND whether they are one- or two-sided<br>*Only common tests should be described solely by name; describe more complex techniques in the Methods section.*
☐	☒	A description of all covariates tested
☐	☒	A description of any assumptions or corrections, such as tests of normality and adjustment for multiple comparisons
☐	☒	A full description of the statistical parameters including central tendency (e.g. means) or other basic estimates (e.g. regression coefficient) AND variation (e.g. standard deviation) or associated estimates of uncertainty (e.g. confidence intervals)
☐	☒	For null hypothesis testing, the test statistic (e.g. F, t, r) with confidence intervals, effect sizes, degrees of freedom and P value noted<br>*Give P values as exact values whenever suitable.*
☒	☐	For Bayesian analysis, information on the choice of priors and Markov chain Monte Carlo settings
☐	☒	For hierarchical and complex designs, identification of the appropriate level for tests and full reporting of outcomes
☐	☒	Estimates of effect sizes (e.g. Cohen's *d*, Pearson's *r*), indicating how they were calculated

Our web collection on statistics for biologists contains articles on many of the points above.

Software and code

Policy information about availability of computer code

Data collection	We extracted data manually from published papers. When the data were presented in a figure, we used Web Plot Digitizer (version 4.2) to read the data. Aridity index data were extracted as described in the Data availability statement in the “Data” box below.
Data analysis	Data analyses were done in the R programming environment (version 4.1.0). We used the metafor package (version 3.0-2) for the meta-analytic mixed-effects models, and to test for publication bias. In each meta-analysis, the MuMIn package (version 1.43.17) was used for making an information-theoretic model selection based on AICc values to identify the minimum adequate model. We used DHARMa package (version 0.1.5) functions for testing overdispersion and homogeneity of residual variances. For each meta-analytic model, we fitted an equivalent linear mixed-effects model using the nlme package (version 3.1-149) to extract ANOVA tables, and computed R-squared values using the r2glmm package (version 0.1.2). We tested whether experimental and observational studies differed in site aridity, drought length, drought severity, and aboveground biomass. For site aridity and drought length we used the glmmTMB package (version 1.1.2.3) and the lme4 package (version 1.1-27.1), respectively, while the differences in drought severity and biomass were tested using the nlme package. The computer code (R scripts) of the analyses is available in Figshare with the identifier https://doi.org/10.6084/m9.figshare.17881073.

For manuscripts utilizing custom algorithms or software that are central to the research but not yet described in published literature, software must be made available to editors and reviewers. We strongly encourage code deposition in a community repository (e.g. GitHub). See the Nature Portfolio guidelines for submitting code & software for further information.

Data

Policy information about availability of data

All manuscripts must include a data availability statement. This statement should provide the following information, where applicable:

- Accession codes, unique identifiers, or web links for publicly available datasets
- A description of any restrictions on data availability
- For clinical datasets or third party data, please ensure that the statement adheres to our policy

The data that support the findings of this study are available in Figshare with the identifier https://doi.org/10.6084/m9.figshare.17881073. Aridity index data were extracted from Global Aridity Index and Potential Evapotranspiration (ET0) Climate Database v2, which is available in Figshare with the identifier https://doi.org/10.6084/m9.figshare.7504448.v3.

Field-specific reporting

Please select the one below that is the best fit for your research. If you are not sure, read the appropriate sections before making your selection.

☐ Life sciences ☐ Behavioural & social sciences ☒ Ecological, evolutionary & environmental sciences

For a reference copy of the document with all sections, see nature.com/documents/nr-reporting-summary-flat.pdf

Ecological, evolutionary & environmental sciences study design

All studies must disclose on these points even when the disclosure is negative.

Study description

We compared the responses of aboveground biomass to experimentally applied versus observed drought events in a systematic review using hierarchical meta-analyses. We tested for the effects of potential confounding factors such as drought severity (% reduction in yearly precipitation), drought length (years), and site aridity (mean annual precipitation divided by mean annual potential evapotranspiration). We used log response ratio (lnRR) as an effect size metric. We weighted data by the number of replications in our focal meta-analysis, but we also conducted additional meta-analyses with different weightings, and for data not used in the focal analysis, to test the robustness of our results.

Research sample

In total, 239 data points were extracted from 114 published papers, and 158 data points of them (from 80 studies) were included in our focal meta-analysis. A data point was a natural or experimental drought event reported in a particular study. Data of different sites, or land use, etc., from the same study were collected as distinct data points, but data points from the same study received a common study ID, and study ID was treated as a random effect in statistical tests. For each study site, we extracted aridity index from Global Aridity Index and Potential Evapotranspiration (ET0) Climate Database v2 (available at https://doi.org/10.6084/m9.figshare.7504448.v3).

Sampling strategy

We conducted a systematic literature search in the ISI Web of Science (WoS; since 1975) for published results on drought effects on aboveground plant production from studies conducted in grasslands or shrublands. For the exact search terms we used please see the Methods section. This yielded 2187 papers, which were screened using the following criteria (established before the start of the screening): The research was conducted in (semi-)natural grasslands or shrublands. The paper reported precipitation reduction relative to the control (non-drought year(s) in observational studies and no treatment in experimental studies), and plant production expressed as aboveground net primary production (ANPP), aboveground plant biomass (in grassland studies only), or percentage plant cover for control and drought. We also included 27 studies meeting these criteria from the references of WoS records and previous reviews. In total, this resulted in 114 studies. Thus, sample size was determined by the number of studies available in the literature worldwide and by our inclusion criteria. Literature search and paper screening were done by G. Kröel-Dulay.

Data collection

From the studies, we collected the study site, latitude, longitude, mean annual temperature (MAT) and precipitation (MAP), study type (experimental or observational), drought length (years), vegetation type (grassland or shrubland), and yearly precipitation for both the control and drought. From precipitation data, we calculated drought severity as % reduction in yearly precipitation in response to drought relative to the control. For production, we compiled the mean, replication, and if the study reported, a variance estimate (standard deviation, standard error of the mean, or 95% confidence interval) for control and drought. Data were extracted from the text, tables or figures of the published papers, and typed into an Excel sheet. When the data were presented in a figure, we used Web Plot Digitizer to read the data. The 114 published papers provided 239 data points. Data collection from the papers was done by A. Mojzes. For each study site, we extracted aridity index from Global Aridity Index and Potential Evapotranspiration (ET0) Climate Database v2.

Timing and spatial scale

We covered the period from 1975 to 13 January 2020 in the WoS search. Additional studies from cited references go back to 1937. Regarding the spatial coverage, we searched for papers from all parts of the world, without any geographic restriction. Since the data were collected from published papers (except for aridity index), the spatial and temporal scales, as well as the frequency and periodicity of sampling were determined by the particular study (these were study specific). Aridity index data covered the period of 1970–2000 (aggregated on annual basis).

Data exclusions

During screening of the papers, we excluded the studies that did not meet our inclusion criteria summarised above in the “Sampling strategy” box. For more details on data exclusion, please see the PRISMA flow chart (Supplementary Fig. 1) and the Methods section. From our focal meta-analysis, we excluded the studies from wet sites, shrublands, or that estimated plant cover, because these were rare and very unequally distributed between experiments and observations (but the excluded data points were analysed separately).

Reproducibility	As our study is a meta-analysis, we did not perform an experiment. The literature search conducted in the WoS database is fully reproducible. For screening of the eligible papers, we set clear criteria for inclusion and exclusion that help reproducibility (see the PRISMA flow chart (Supplementary Fig. 1) and the Methods section). We provide the data and R code required to repeat the analyses we performed (available at https://doi.org/10.6084/m9.figshare.17881073).
Randomization	Randomisation is not really relevant in our study as we worked with data found in the literature, and the design of the original studies clearly defined if a study (drought) is experimental or observational. However, we accounted for three potential confounding factors (site aridity, drought length, and drought severity) by including them as predictors in the statistical models, and used study ID as a random effect. In addition, we found no evidence of publication bias when testing either the whole data set included in the focal meta-analysis, or experimental and observational studies separately.
Blinding	Blinding is not relevant in our study, because we extracted data from published studies. The design in each study determined the study type (i.e. experimental or observational), so it was not possible to blind ourselves whether a study is observational or experimental.

Did the study involve field work? ☐ Yes ☒ No

Reporting for specific materials, systems and methods

We require information from authors about some types of materials, experimental systems and methods used in many studies. Here, indicate whether each material, system or method listed is relevant to your study. If you are not sure if a list item applies to your research, read the appropriate section before selecting a response.

Materials & experimental systems

n/a	Involved in the study
☒	☐ Antibodies
☒	☐ Eukaryotic cell lines
☒	☐ Palaeontology and archaeology
☒	☐ Animals and other organisms
☒	☐ Human research participants
☒	☐ Clinical data
☒	☐ Dual use research of concern

Methods

n/a	Involved in the study
☒	☐ ChIP-seq
☒	☐ Flow cytometry
☒	☐ MRI-based neuroimaging
