# Supplementary material for: Field experiments underestimate aboveground biomass response to drought
Source: Nat Ecol Evol. 2022 Mar 10;6(5):540–5. doi: 10.1038/s41559-022-01685-3 (PMC9085612; doi:10.1038/s41559-022-01685-3)
Supplement: Supplementary file 3 — Peer Review Information [file 41559_2022_1685_MOESM3_ESM.pdf]

Peer Review Information

Journal: Nature Ecology & Evolution
Manuscript Title: Field experiments underestimate aboveground biomass response to drought
Corresponding author name(s): György Kröel-Dulay

Editorial Notes:

Reviewer Comments & Decisions:

Decision Letter, initial version:

1st October 2021

*Please ensure you delete the link to your author homepage in this e-mail if you wish to forward it to your co-authors.

Dear Dr Kröel-Dulay,

Your manuscript entitled "Field experiments underestimate aboveground biomass response to drought" has now been seen by three reviewers, whose comments are attached. The reviewers have raised a number of concerns which will need to be addressed before we can offer publication in Nature Ecology & Evolution. We will therefore need to see your responses to the criticisms raised and to some editorial concerns, along with a revised manuscript, before we can reach a final decision regarding publication.

In particular, we are concerned by Reviewer 2's points about the observational and experimental systems representing different community types (i.e. with different species richness), which complicates the conclusions. We will expect to see additional or modified analyses to address this point and the other points raised by the reviewers.

We therefore invite you to revise your manuscript taking into account all reviewer and editor comments. Please highlight all changes in the manuscript text file.

We are committed to providing a fair and constructive peer-review process. Do not hesitate to contact us if there are specific requests from the reviewers that you believe are technically impossible or unlikely to yield a meaningful outcome.

When revising your manuscript:

* Include a “Response to reviewers” document detailing, point-by-point, how you addressed each reviewer comment. If no action was taken to address a point, you must provide a compelling argument. This response will be sent back to the reviewers along with the revised manuscript.

* If you have not done so already please begin to revise your manuscript so that it conforms to our Brief Communication format instructions at http://www.nature.com/natecolevol/info/final-submission. Refer also to any guidelines provided in this letter.

* Include a revised version of any required reporting checklist. It will be available to referees (and, potentially, statisticians) to aid in their evaluation if the manuscript goes back for peer review. A revised checklist is essential for re-review of the paper.

Please use the link below to submit your revised manuscript and related files:

[REDACTED]

Note: This URL links to your confidential home page and associated information about manuscripts you may have submitted, or that you are reviewing for us. If you wish to forward this email to co-authors, please delete the link to your homepage.

We hope to receive your revised manuscript within four to eight weeks. If you cannot send it within this time, please let us know. We will be happy to consider your revision so long as nothing similar has been accepted for publication at Nature Ecology & Evolution or published elsewhere.

Nature Ecology & Evolution is committed to improving transparency in authorship. As part of our efforts in this direction, we are now requesting that all authors identified as ‘corresponding author’ on published papers create and link their Open Researcher and Contributor Identifier (ORCID) with their account on the Manuscript Tracking System (MTS), prior to acceptance. ORCID helps the scientific community achieve unambiguous attribution of all scholarly contributions. You can create and link your ORCID from the home page of the MTS by clicking on ‘Modify my Springer Nature account’. For more information please visit please visit www.springernature.com/orcid.

Please do not hesitate to contact me if you have any questions or would like to discuss these revisions further.

We look forward to seeing the revised manuscript and thank you for the opportunity to review your work.

[REDACTED]

Reviewer expertise:

Reviewer #1: Grassland drought responses

Reviewer #2: Grassland drought responses

Reviewer #3: Meta-analysis

Reviewers' comments:

Reviewer #1 (Remarks to the Author):

Review of "Field experiments underestimate aboveground biomass response to drought"
In this study, Kröel-Dulay and co-authors investigate to which extent field studies are suitable for studying how aboveground biomass responds to drought, and what biases may or may not exist. They find that drought responses in field experiments are only half as large as responses to natural droughts, implying that current experiments have strong shortcomings. In general, I found the study well conducted, and I appreciate that the authors have attempted to have an important, but also nuanced message, in that they mention the benefits of experiments, despite their limitations. That said, I also had some concerns.
First, while the authors conclude that experiments tend to underestimate drought effects, one could also argue the other way around, namely that observational studies tend to overestimate drought effects. As the authors mention, droughts almost never come alone, but are often accompanied by other extreme events, such as extremely high temperatures. In such cases, it is possible that what observational studies infer as drought effects are in fact effects of other, correlated factors, such as heatwaves. I think the study would be a bit more balanced if the authors also reflect on this possibility.
I found the introduction of this manuscript a bit 'dry' (no pun intended). The introduction is rather methodological, but would be more interesting if also more attention was spent on ecological reasons on why effects of drought may differ between experiments and observational studies. For example, the plots of many drought experiments are often a few square meters, and I could imagine that edge effects (which buffer the treatment effects) play a large role. It would be good to reflect on this or other points that lead to more specific-ecology driven hypotheses.
I also think the authors could do a better job in describing the novelty of their study. In this respect, the introduction was a bit unclear: at some point, the authors mention that no other study has compared the drought effects between observational and experimental studies using a meta-analysis, whereas elsewhere, they seem to imply that only 'few' of such studies exist. Clarity on this is key for understanding what the main novelty of the current manuscript is.
More detailed comments:
L 73-74 "synthesis studies have not compared these two approaches": this seems to somewhat contrast with "A recent overview of ecological responses to global change found that an overwhelming majority of meta-analyses covered either experimental or observational case 65 studies, while only three out of 36 assessed both types." (L63-65) and is a bit confusing for naive readers: on the one hand, I get the impression that meta-analyses comparing observational and experimental do not exist, and on the other hand, I get the impression that such meta-analyses are perhaps rare, but do nevertheless exist. It's important to be clear on this aspect, as it has implications for the novelty of the current study.
Line 100-101: I assume this is after correcting for confounding factors, such as drought severity, duration, etc, right? Although the paragraph before suggests so, it's good to be explicit about this, so that readers can assess whether the comparison is as fair as possible.
L 101: "positively related to": since effects of drought on biomass are typically negative, I am not sure

whether “positively related to” in this context relates to less negative, or more negative. Less/more negative would be clearer wording, less prone to mis-interpretation.
Line 162-163: “Compared to experimental studies, observational studies report stronger effects of warming on ... aboveground biomass”: while the authors cite a meta-analysis in support of this, the given meta-analysis was based on a very small fraction of the literature that is actually available, and some other reviews point at a more nuanced picture, with plant-diversity-biomass productivity relationships being rather mixed in observational studies.
Line 171: “When driving variables are correlated, as often happens in nature, effects of individual drivers are difficult to disentangle”: this is a good point, and leads to a question that I think is underexplored in this study: does the finding that relationships between drought responses of AGB are stronger in observational studies than in experiments mean that experiments underestimate effects, or does it indicate that observational studies overestimate effects? (note that these hypotheses are not mutually exclusive). Correlated extreme events, such as drought coupled with high temperatures, may leads to overestimations of drought effects in observational studies, if observed correlations re actually driven more by temperature than drought per se.
PRISMA flow diagram: I saw that one study was excluded because it assessed precipitation exclusion in winter. In some climates (e.g. in eastern South Africa) winter is the main dry season. It would be good to re-check the study, and only exclude it if winter was relatively irrelevant for studying droughts.
ESM1: Since one other study “Studies synthesising both observational and experimental droughts” is listed, it would be good if it becomes clear how the current manuscripts differs from this existing work.
ESM2: personally, I think it’s good if these studies are cited in the main text as well (not only in the supplement), so that the authors of the original studies get credit for their work.

Reviewer #2 (Remarks to the Author):

This is a nicely succinct study that tackles an important question – do experiments underestimate the effect of drought on plant biomass? While the body of the paper is very well written and the implications of this study seem important, there are a few potentially fundamental flaws in the analyses and representation of the data that need to be addressed. In addition, there are a number of details that have been omitted in the methods, results, and figures that make interpretation difficult. I have outlined my main concerns and the additional details that will be critical to address in order to support the bold claim of this paper.

Reviewer #3 (Remarks to the Author):

The paper by Kröel-Dulay and Mojzes et al. asks whether analysing the effects of drought on terrestrial plants leads to different results when relying on observation or experimentation. To answer this question they meta-analyse across different study types and take a range of potentially important covariates into account. They conclude that experiments systematically underestimate drought effects.

Being not a terrestrial ecologist I was asked to especially have a look on the conduction of the meta-analysis in this manuscript. I am very positive about the handling and reporting of the search, data handling, statistics and sensitivity analyses. The authors exemplarily follow the guidelines for reproducible synthesis and whenever I thought about a potential analyses needed to test for the sensitivity of decisions made, I found these in the supporting material.

Still I have a few comments that need to be addressed.

1) Why only aboveground biomass or NPP? I am pretty sure that other response variables have been measured frequently in these experiments and it would be very informative to see whether the observed differences are general across responses – is it just ANPP or are other processes less sensitive to the experimentation.

2) I agree that the size of the treatment application is a potential cause here. Drought experiments dry a small area of soil in a wet surrounding, whereas real droughts dry up larger regions. Thus, lateral flows of water and edge effects might not at all be important in observations, but surely are in the m^2 range of experimental plots. While this is somewhat discussed in the paper I wondered i) why it is not be made more explicit (row 148ff talks about a lot of differences, but does not make the main difference very clear) and ii) if there would not be a way to directly test this - at least experiments of different size must exist and it may be possible to also estimate the spatial extent of observed droughts.

3) The authors argue, and bring some evidence, that experiments might underestimate the magnitude of global change effects. However, this is less general than stated here. Experiments tend to overdo the differences between treatments and control (see. E.g. Korell et al. 2020, Global Change Ecology) and have secondary crowding effects (Moise & Henry 2010 Oikos, Hillebrand 2009 J Phycol). The Korell paper is so close to the topic here that it should be included in the discussion. Moreover, I would like to see the discussion more nuanced and shifted towards why drought experiments may be especially prone to this underestimation.

********************END********************

Author Rebuttal to Initial comments

Reviewers' comments:

Reviewer #1 (Remarks to the Author):

Review of “Field experiments underestimate aboveground biomass response to drought”
In this study, Kröel-Dulay and co-authors investigate to which extent field studies are suitable for studying how aboveground biomass responds to drought, and what biases may or may not

exist. They find that drought responses in field experiments are only half as large as responses to natural droughts, implying that current experiments have strong shortcomings. In general, I found the study well conducted, and I appreciate that the authors have attempted to have an important, but also nuanced message, in that they mention the benefits of experiments, despite their limitations.

R.2. We very much appreciate the positive words of the Reviewer regarding our manuscript, in particular on the way we tried to offer a balanced picture on the pros and cons of field experiments. Please find our point by point responses.

That said, I also had some concerns.
First, while the authors conclude that experiments tend to underestimate drought effects, one could also argue the other way around, namely that observational studies tend to overestimate drought effects. As the authors mention, droughts almost never come alone, but are often accompanied by other extreme events, such as extremely high temperatures. In such cases, it is possible that what observational studies infer as drought effects are in fact effects of other, correlated factors, such as heatwaves. I think the study would be a bit more balanced if the authors also reflect on this possibility.

R.3. We agree that this is an important issue and we considered this already in the writing process, and also during this revision. When a natural drought event occurs, it encompasses more than just below-avarege precipitation. However, these accompanying factors (higher temperature, clear sky, low air humidity) are generally viewed as intrinsic aspects of natural drought events (De Boeck and Veerbeck 2011). This is why we argue that experiments that simulate only less precipitation but not other elements of drought, are underestimating drought effects, rather than observational studies on natural drought overestimating them. In the revised version, we more explicitly address this:

„A potential reason for underestimation of drought effects in experiments could be that they simulate less rain, but do not control for increased evaporative demand associated with high temperatures, low humidity, and clear skies. Given that droughts in reality are typically accompanied by these intensifying factors[17], we assert that drought experiments underestimate drought effects as manifested in nature, rather than that observational studies overestimate them."

I found the introduction of this manuscript a bit 'dry' (no pun intended). The introduction is rather methodological, but would be more interesting if also more attention was spent on ecological reasons on why effects of drought may differ between experiments and observational studies. For example, the plots of many drought experiments are often a few square meters, and I could imagine that edge effects (which buffer the treatment effects) play a large role. It would be good to reflect on this or other points that lead to more specific-ecology driven hypotheses.

R.4. We indeed do not provide much detail on ecological background behind possible differences, but what triggered our paper was: (1) there are many experimental and observational case studies, but very few across-type comparisons in environmental change context, and (2) the complete lack of such synthetic comparisons on droughts. Also, please note that the Brief Communication format allows only limited length, and with the changes in this revised version we are already a little above the suggested length. Hence, we prioritized focusing on the core facts relating to this research gap and its potenial importance. Nevertheless, we now added half a sentence at the end of the second paragraph in order to specify factors that may explain differences between experimental and observational results:

"This bias towards experimental drought studies is concerning in light of the limitations of climate change experiments, such as small spatial extent[2] and inability to replicate the full set of naturally occurring drought conditions[1]."

I also think the authors could do a better job in describing the novelty of their study. In this respect, the introduction was a bit unclear: at some point, the authors mention that no other study has compared the drought effects between observational and experimental studies using a meta-analysis, whereas elsewhere, they seem to imply that only 'few' of such studies exist. Clarity on this is key for understanding what the main novelty of the current manuscript is.

R.5. Thanks for this comment; it is really important to make clear what is new in our study. Comparison of experimental and observational results in meta-analyses are very rare in studies on environmental change (we highlight this in the first paragraph), and it is non-existent for droughts. We now tried to further highlight the latter by rewording the related sentence in the second paragraph:

„ Although many observational and experimental studies have assessed the effects of drought events, no synthesis study on droughts has compared results from these two approaches".

More detailed comments:

L 73-74 “synthesis studies have not compared these two approaches”: this seems to somewhat contrast with “A recent overview of ecological responses to global change found that an overwhelming majority of meta-analyses covered either experimental or observational case studies, while only three out of 36 assessed both types.” (L63-65) and is a bit confusing for naive readers: on the one hand, I get the impression that meta-analyses comparing observational and experimental do not exist, and on the other hand, I get the impression that such meta-analyses are perhaps rare, but do nevertheless exist. It’s important to be clear on this aspect, as it has implications for the novelty of the current study.

R. 6. We first (l.63-65, first paragraph) write about meta-analyses on environmental change effects in general, where a few comparisons do exist. We then, in the second paragraph, narrow the focus to the domain of drought studies, where no such synthesis study exists. We modified the sentence on droughts to make this dictinction clear (pease see response R.5. above).

Line 100-101: I assume this is after correcting for confounding factors, such as drought severity, duration, etc, right? Although the paragraph before suggests so, it’s good to be explicit about this, so that readers can assess whether the comparison is as fair as possible.

R.7. We originally presented the actual difference between the means of observational and experimental results, but we agree that reporting the corrected (modelled) values would be more consistent with the statistical analysis. We now report the difference between the estimated means based on the coefficients of the final minimum adequate model shown in ESM 7. In fact, this difference became even bigger after correcting for the additional factors. For consistency, we now present these modelled means also on Figure 1, and include confidence interval in both the text and the figure, following the suggestion by Reviewer 2 (R.23.). In order to make clear what this number means, we changed the text as follows: "

„The estimated mean effect of drought was 53% (CI: 90; 16) weaker in experimental than observational studies, after controlling for potentially confounding factors (Fig. 1, ESM 7).”

L 101: “positively related to”: since effects of drought on biomass are typically negative, I am not sure whether “positively related to” in this context relates to less negative, or more negative. Less/more negative would be clearer wording, less prone to mis-interpretation.

R.8. Thanks for highlighting this wording for potential misundersanding. We now reworded this sentence as follows: „Drought responses increased with increasing aridity and, marginally, with increasing drought severity…”

Line 162-163: “Compared to experimental studies, observational studies report stronger effects of warming on ... aboveground biomass”: while the authors cite a meta-analysis in support of this, the given meta-analysis was based on a very small fraction of the literature that is actually available, and some other reviews point at a more nuanced picture, with plant-diversity-biomass productivity relationships being rather mixed in observational studies.

R.9. We agree that there are controversies around the issue of species richness – productivity relationships, especially in natural settings (observational studies). We therefore decided remove this example from the list.

Line 171: “When driving variables are correlated, as often happens in nature, effects of individual drivers are difficult to disentangle”: this is a good point, and leads to a question that I think is underexplored in this study: does the finding that relationships between drought responses of AGB are stronger in observational studies than in experiments mean that experiments underestimate effects, or does it indicate that observational studies overestimate effects? (note that these hypotheses are not mutually exclusive). Correlated extreme events, such as drought coupled with high temperatures, may leads to overestimations of drought effects in observational studies, if observed correlations re actually driven more by temperature than drought per se.

R.10. We carefully considered this comment, and reworded this part of the discussion to give a more balanced picture and justify our opinion. Please see R.3. for a detailed response on this issue.

PRISMA flow diagram: I saw that one study was excluded because it assessed precipitation exclusion in winter. In some climates (e.g. in eastern South Africa) winter is the main dry

season. It would be good to re-check the study, and only exclude it if winter was relatively irrelevant for studying droughts.

R. 11. We checked the paper we excluded for the reason „winter precipitation exclusion". In this paper, the study site (close to Boulder, Colorado, USA) has a continental climate, with most precipitation falling in spring and early summer, and the growing season is usually from March to October. In the reported experiment, decreased precipitation treatment was applied from early October to late March, i.e. outside the growing season. As we did not include experiments in which drought was applied outside the growing season (Detailed Methods, ESM 6), we kept this study excluded from our dataset. However, we replaced "precipitation exclusion in winter" by "precipitation exclusion outside the growing season" in the PRISMA flow diagram (ESM 2).

ESM1: Since one other study "Studies synthesising both observational and experimental droughts" is listed, it would be good if it becomes clear how the current manuscripts differs from this existing work.

R.12. This particular synthesis covers both experimental and observational case studies, but it does not compare the two study types. In order to make this clear, we changed the title to:

„Studies covering (but not comparing) observational and experimental droughts"

ESM2: personally, I think it's good if these studies are cited in the main text as well (not only in the supplement), so that the authors of the original studies get credit for their work.

R.13. We fully agree that it would be nice if case studies covered by a meta-analysis would get credit, at least in a way of being listed in the Reference list. In our understanding, however, the usual way these days is to include such lists in an Electronic Supplementary Material.

Reviewer #2 (Remarks to the Author):

This is a nicely succinct study that tackles an important question – do experiments underestimate the effect of drought on plant biomass? While the body of the paper is very well written and the implications of this study seem important, there are a few potentially fundamental flaws in the analyses and representation of the data that need to be addressed. In

addition, there are a number of details that have been omitted in the methods, results, and figures that make interpretation difficult. I have outlined my main concerns and the additional details that will be critical to address in order to support the bold claim of this paper.

R.14. We very much appreciate the detailed evaluation of our manuscript, and the positive words on the importance of the implications. We carefully went through the comments and suggestions, and added our responses below.

Major comments:
1. My biggest concern is that experimental and observational studies are not matched by community type. Ideally this is what you should be comparing – experimental and observational studies from the same area.

R.15. We absolutely agree with the importance of this issue and that the best approach would be to pair data points from the same stites. Unfortunately, we found no studies of this type except for Knapp et al., already cited. To highlight the issue, we now explicitly included the need for more direct comparisons in the discussion as a potential further step to elucidate differences between experiments and observations

„Further work is needed to test the generality of the observed discrepancies between experimental and observational results, and this should include both systematic comparison of study types across global change factors and matched case studies, where observational and experimental results come from the same sites."

In the absence of paired sites, we focused on making the realm of the two kinds of studies (experimental vs. observational) similar, and applied several steps to ensure this as far as possible.

(a) We included only field experiments in natural/seminatural habitats, because we think that experiments in these habitats can be compared more straightforwardly to natural drought events (in natural/seminatural habitats) than drought events in experimentally constructed communities. As the reviewer points out, species richness can potentially be lower in experiments. However, this may be true for artificial communities (pot experiments, sown grasslands, etc.), but is less likely to be the case in natural communities, i.e. the same types as where natural drought events occur.

(b) We left out from the focal analysis the data where data points were unequally distributed between the two types of studies (shrublands, wet sites, and grasslands with cover values), as this could have biased the analysis. Note that we also tested these extra points, but in a

separate analysis, with a similar outcome and thus reinforcing the robustness of our conclusions.

(c) We tested if experimental and observational studies differ in their abiotic environment, which can also be viewed as a determinant, or even a proxy, of community type. We used an aridity index (measured as Mean Annual Precipitation divided by Mean Annual Potential Evapotranspiration) for this purpose, and found no difference between observational and experimental studies. See also R.16, where, in response to the comment by the Reviewer, we also compared aboveground biomass of the two types of studies.

Biomass/productivity is often correlated with plant diversity (e.g., Hooper et al., 2012; https://www.nature.com/articles/nature11118). If there is a systematic bias of plant diversity in study type (i.e., experimental studies typically include less species), then this could be driving your results – not the experimental efficacy. Either including species richness as a covariate or weighting by richness could help control for this potentially confounding factor.

R.16. We considered this suggestion carefully, and checked if and how we could test if species richness is different between experimental and observational studies. Please note that we only included experiments (and also observational studies) that took place in natural/seminatural environments, and thus species richness should not be lower in these experimental studies compared to observational studies (see. also R. 15). In order to test this, we searched the papers that were included in the focal data set for species richness data. However, we found data on species richness only in 16 out of 80 papers (20%), and even these data were from plots of very different size. This low number, as well as the variable plot sizes, did not make it possible to include this variable in the analysis as a potential confounding factor, or even to reliably compare experimental and observational studies in a separate analysis. We now added this information in the Detailed methods (ESM 6) as follows:

"In addition, we considered two other potential confounding factors: plant species richness, ... and dominant life form .... However, we found very limited species richness data; it was included in 16 studies only (20% of studies). Furthermore, these data were estimated at various spatial scales (ranging from 0.04 to 10000 m^2) depending on the study. Therefore, we could not include species richness in the analysis as a potential confounding factor, or even reliably compare this variable between the two study types in a separate analysis."

However, in line with the argumentation of the reviewer that species richness and community biomass are often correlated, and that community biomass can also be a proxy of community type, we compared aboveground biomass in experimental and observational studies. We found

no difference in aboveground biomass between the two study types in our focal dataset (ESM 11). We included this information also in the manuscript:

„site aridity, drought severity, and aboveground biomass (control) were similar in experimental and observational studies"

In conclusion, even if the lack of data did not allow to include species richness in our analysis, we think that (1) careful selection criteria (natural settings only), (2) exclusion of community types with unequal representation in the two study types (wet sites, shrublands) from our focal data set, and (3) testing of variables that may serve as proxies for community type (aridity, aboveground biomass) further ensure that the two study types covered were comparable, with limited room for bias.

2. My second major concern lies in the modelling approach. It's surprising that study type is not significant in any of the full models. The full models do not seem overfit and by selecting the most "parsimonious" models without showing what the differences in AIC magnitude are – this approach could lead to misleading results and/or increased type II error. Selection of variables should be conducted via AIC rather than p-values. In addition, by reducing the number of explanatory variables in your model via AIC, you are potentially decreasing the interpretability of each coefficient. Selection via AIC is useful for prediction purposes – it's much less useful if the aim is to identify or describe mechanisms/underlying processes (see Galipaud et al., 2017, Methods in Ecology; **https://doi.org/10.1111/2041-210X.12835**). In summary, your final model used for the analysis presented in the paper is currently poorly justified and implemented. Further justification of your model selection approach is necessary to support your bold conclusions about study type.

R.17. Thank you for this important comment on our modelling approach, and, in particular, highlighting the striking difference between the full model and minimum adequate model, which makes the model selection procedure critical. We realised that we erroneously named the parameter estimate table an ANOVA table in the previous version (this parameter estimate table is the standard output in the metafor package). This was confusing because the P-values in this parameter table do not correspond to the significance of F-tests for predictors, but show the statistical significance of each coefficient of the model. To avoid this confusion, in the new version, we show both the ANOVA table of the models and the model parameter estimate table. Please note that these parameter tables also changed compared to the previous version, because following the suggestion of statisticians, the three continuous variables were centred to avoid multicollinearity and to get easily interpretable parameter estimates (see Methods and ESM 6).

Looking at the ANOVA tables, the full models and the minimum models are very similar. Please see the details of the analyses in ESM 7-9.

Regarding the issue of model selection, thank you for suggesting the paper of Gallipaud et al. 2017. There is a lively discussion in the ecological statistical literature on the optimal explanatory modelling approaches to reveal the drivers of biological mechanisms (Shmueli 2010 in Statistical Science). The use of full models containing all possible predictors (Wittingham et al. J of Animal Ecol. 2006), the selection of a single best model (e.g. Emiliano et al. 2014, Computational Statistics ad Data Analysis, Zuur et al. 2009, Mixed-effects Modelling and Extensions in R), or a subset of models are all advocated for exploratory purposes (e.g. Gallipaud et al. 2017). There are a number of papers that emphasize the importance of parsimony and therefore suggest trying to find the simplest model that best describes the data, while others feel that more complex models are always more desirable (Aho et al. 2014, Ecology).

After a thorough discussion with our statistical expert, we re-ran our modelling so that we focused on finding the most parsimonious model based on information-theoretic analysis to avoid identifying spurious variables as important and overfit models (Johnson & Omland, TREE 2004). We used AICc instead of AIC values because of small sample size advocated by Sugiura (1978; Communications in Statistics) when N< K/40. We searched for the model with minimum AICc from all the possible models nested in our full model and used this for presenting the final conclusions about the effect size moderation of our predictors. However, we also included the results of the full models for reference in ESM 7-9. The AICc and also AIC values of the models are given in the table legends in ESM 7-9 to show that the minimum model had much lower AICc values in every case compared to the full models.

We included this modelling approach in the Detailed Methods (ESM 6):

"Besides the full model in each meta-analysis, we made an information-theoretic model selection based on AICc values by using the dredge function of the MuMIn package (Bartoń 2020) to identify the minimum adequate model that is best supported by the data (Johnson & Omland 2004)."

In conclusion, thank you for highlighting the discrepancy between the full models and the minimum adequate models, and the issue of model selection. When looking at the ANOVA tables (instead of the previously presented parameter estimate tables), the differences between the full and minimum models are limited, and we decided to stick to the minimum adequate models, based on the argumentation above.

3. The premise and arguments are often extrapolated beyond the data and should be furtherspecified, as there is no way to know if drought experiments consistently underestimate drought impacts across multiple biomes or even the grassland biome. Please reduce phrasing throughout your manuscript that appears to extrapolate to all systems and caveat your statements with “semi-arid grasslands” or similar phrasing.

R.18. During our literature search we looked for studies in all kinds of grasslands (from desert grasslands to mountain meadows excluding wetlands) and shrublands, and related ecosystems (described in detail in ESM 6). Admittedly, in our detailed analyses with several moderators, we excluded shrublands and wet grasslands (ESM 7-9), in order to have a more balanced data set. However, in a separate analysis (ESM 10) we also analysed these excluded points, and found a similar difference between experiments and observations, reinforcing our main conclusion. Also, based on this additional analysis, we think that our most important result that responses are smaller in experiments than in observations is valid across a broader domain of ecosystems than just semiarid grasslands. We now included a sentence to highlight this:

„ This latter finding suggests that the general pattern of weaker response in experiments holds beyond grasslands (focal data set), even if the low number and unequal distribution of studies did not allow for a detailed analysis across a broader range of ecosystems.”

However, we agree that we should still mention this limitation in the discussion, so we added this specification to the beginning of the discussion part.

„Our findings suggest that experiments significantly underestimate the effects of droughts in grasslands and shrublands.”

In addition, I’d like to see a
figure showing where these studies were implemented (color denoting observational and experimental) and the climate variability of the ecosystems represented in this study -- i.e., you state you exclude wet sites – so what proportion of the grassland biome is represented in this study?

R.19. We accepted this suggestion and present the geographic distribution of studies on a map (ESM 5). Please note that our analyses also covered wet grasslands and shrublands, so the data could represent the full grassland biome and beyond (see also R.18 on this issue). We think that a biome map or climate map as a background would complicate the map, and could decrease the visibility of data points, so we just put study locations on a plain map of continents.

Minor comments:
Abstract:

1. The abstract needs more details of the methods and results to be interpretable.
R.20. Please be aware that there is a strict limit on the length of the abstract in Brief Communications (max. 3 sentences and max 100 words). We thought that one (first) sentence should introduce the general problem, one sentence (third) should offer an overall message, and thus we had one sentence left for Methods and Results. Nevertheless, we added some more methodological details:
„ Using a meta-analysis and accounting for potential confounding factors, we demonstrate that…"

Methods and results:
1. You wrote: "If it was not specified in the paper, the year immediately preceding the drought year(s) was chosen as control. When no data from the pre-drought year was available, the year immediately following the drought year(s) (14 data points), or a multi-year period given in the paper (22 data points) was used as control." a. Including just one year as the control is potentially problematic if that year does not capture average climatic conditions. Please verify that these data points (with only one year of control) are not driving your results.

R.21. We chose one year preceding (or following) the drought year(s) as control for the following reasons:

a) We believe that the plant production of the drought year shows the effect of drought most reliably when compared to the production of immediately preceding (or following) year, as the vegetation is not likely to change from one year to the next due to other factors (e.g. succession) than drought.

b) We could use this criterion consistently (apart from some exceptions mentioned in the Detailed methods, ESM 6).

c) In many cases, only one-year data were available in the papers for comparison with the drought year.

d) Wetter or drier-than-average year may also be a confounding effect in drought experiments, especially when they last for only one year.

Nevertheless, we now checked in the focal data set how the control year or years we used capture the average precipitation of the study site by calculating the percentage difference between the control year and the mean annual precipitation (MAP) of the study site. We found that the percentage deviation of control precipitation from the respective MAP did not exceed ± 20% in 39 out of the 41 studies. Based on these reasons, we do not think that using one year as control would cause systematic bias.

2. “For production (i.e. response variable), two types of effect size metric were calculated: log response ratio (lnRR) and percent change relative to control.” a. % change could increases with site aridity (unless biomass iis standardized) simply because these sites are on average less productive. So I’d just use your lnRR results, unless there’s another reason to keep both.

R.22. % change was measured as relative to control (so it was standardised), but this other metric did not really add new insights, which is why we decided to leave this out completely, as suggested by the Reviewer.

3. Line 100: “The mean effect of drought was 45% weaker in experimental than observational studies” – this statistic needs to capture the uncertainty of the two distributions – please add CI for this statistic.

R.23. We added confidence intervals to the mean to give an estimation of uncertainty as requested. Please note that we now have a slightly different number (an even stronger response: 53% instead of 45%), because following the comment of Reviewer 1 (R.7) we now report the model estimate, which is already controlled for confounding factors. We also added confidence interval estimation to each parameter estimate of the meta-analytic models in ESM 7-10 and 13.

4. You mention 4 additional robustness checks for the metanalysis but don’t clearly reference where to find the results -- please clarify and present these results in the supplement.

R.24. We now report on three additional meta-analyses with different weightings (variance-weighted and unweighted), and on data excluded from the focal analysis, because following your suggestion we left out the use of percent change as a second effect size metric. We refer to the supplementary material for the detailed results of these analyses in the Results section, where we report their major findings (ESM 8-10). In order to make the link between the respective method and result sections more clear, we now use the same wording, „additional meta-analysis”.

5. All tables should include: R2, AIC, and number of observations

R.25.We complemented the table legends of the parameter estimates in the Supplementary materials with AICc and AIC values, R-squared values calculated according to Nakagawa & Schielzeth (Methods in Evol Evol, 2013) and with the number of samples.

6. Please add funnel plots to the supplement

R.26. We added three funnel plots of the experimental, the observational, and the whole focal dataset for reference in ESM 12 as requested.

7. Lagged drought effects likely vary by functional group/life form (annual/perennial/mixed). If experimental studies are systematically biased towards perennial species but observational studies capture both annuals and perennials, then it will be critical to standardize your studies by life form.

R.27. This is a good point, so we now checked the dominant life form in the studies included in our focal meta-analysis. However, we found that most (87.5% of) studies were conducted in perennial grasslands, and only a few of them (only 10 studies out of 80 studies) were performed in annual grasslands. We supplemented the Detailed methods (ESM 6) with these details as follows:

"In addition, we considered two other potential confounding factors: plant species richness, ... and dominant life form (annual vs. perennial), because annual-dominated ecosystems may be less resistant to drought than those dominated by herbaceous perennials (Ruppert et al. 2015)."
And:
"Regarding dominant life form, the overriding dominance of perennial grasslands in both study types (70 studies out of the 80 studies of our focal dataset) did not allow us to include this variable in our analysis."

8. "artificially constructed plant communities or studies using monolith transplants were excluded" What does artificially constructed mean exactly? Were studies included that transplanted multiple species? Please add more clarifying details.

R.28. Because studies on natural drought events typically report drought effects on intact ecosystems, we considered drought experiments to only be comparable to natural droughts if we restrict our search to similarly intact ecosystems subjected to experimental drought. Transplants, even if they are multispecies transplants, would differ in several ways (e.g. rooting depth, edge effects) and would thus be less comparable to natural (observational) drought events. In order to justify this restriction we added a sentence in the Detailed Methods (ESM 6).

„We used this restriction because most reports on observational droughts are from intact ecosystems, and experiments in disturbed sites or using artificial communities would thus not be comparable to observational drought studies."

Artificially constructed means „seeded" or „planted"; we now added this specification in the text in the Detailed methods (ESM 6).

9. Please provide the N for the number of studies using ANPP/biomass/%cover in observational vs experimental studies.

R.29. We collected studies that reported production data expressed in either aboveground net primary production (ANPP), aboveground plant biomass (in grassland studies only), or percentage plant cover. However, in our meta-analyses we did not distinguish between ANPP and aboveground biomass (see below), thus we used two production variables: biomass and cover (as we mentioned in the Detailed methods, ESM 6), and in the focal data set (grasslands) we only had aboveground biomass.

We now reworded the respective part of the methods section (ESM 6):
"In 79% of studies that used ANPP as a production variable and we selected for our meta-analyses, ANPP was estimated by harvesting peak or end-of-season aboveground biomass. Therefore, we did not distinguish between ANPP and aboveground biomass, and referred to as "biomass" hereafter."

Our table of the collected data (ESM 4) includes a "Production variable" column that indicates whether production was estimated either by biomass or cover for each data point. This constitutes one of the differences between the focal and the additional dataset (i.e. our focal dataset includes studies with biomass data only, while studies that estimated cover are used in the additional data set only).

10. How correlated were your explanatory variables used in the full models – please provide a correlation table.

R.30. Thank you for this suggestion. We now checked for multicollinearity using variance inflation estimation. We preferred this method as this can describe multiple correlations beside pairwise correlations. We provide these values in ESM 7-9. All VIF values were below 3 (except for a single interaction term (3.11); ESM 7), which indicates that no multicollinearity issues were present in the models.

Discussion:

11. Please provide a discussion of the caveats of your study – clearly there are limitations in the # of studies, this study is not matched by grassland type or spp. diversity, you are combining a number of different types of "biomass" estimates, you are excluding a number of studies,

etc.

R.31. We agree that our study has caveats. Please note that we excluded studies from our focal analysis only, but we used them in a separate analysis.

We discuss issues that limit more detailed analyses:

„In practice, using a drought severity metric that incorporates not only precipitation reduction but also variables such as temperature, humidity, and cloud cover could narrow the gap between experimental and observational results. However, infrequent reporting of these variables in individual studies hinders such analyses."

We now included additional limitations:

„ the low number and unequal distribution of studies did not allow for a detailed analysis across a broader range of ecosystems".

Furthermore, we added a sentence on how future studies could better assess the generality of the difference between experimental and observational results:

„Further work is needed to test the generality of the observed discrepancies between experimental and observational results, and this should include both systematic comparison of study types across global change factors and matched case studies, where observational and experimental results come from the same sites.."

12. Lines 174-176: "observations to estimate the "real" net effects of climate change in realistic settings including all interacting factors, and experiments to test causation and reach a mechanistic understanding."
a. This statement seems to contradict your main message that experimental studies are not capturing their intended effect – the causal pathway of drought on biomass. In previous sections of the discussion, you suggest that it's unclear how to interpret the efficacy of drought experiments. For example, are rainout shelters just capturing the causal effect of less rain? Could there be more experimental interference than previously thought leading to the discrepancy? Please clarify what you mean here.

R.32. Yes, this statement was probably not entirely clear in its original format. Experiments can test cause-effect relationship, but only for the actual treatment/manipulation that they include, which may or may not be the same as (or very close to) what they are intended for.

We reworded the particular sentence:
„and experiments to test causation for clearly defined and experimentally reproducible driving variables and thereby obtain a mechanistic understanding"

Yes, drought experiments probably simulate less rain, but not other features of drought. They can still test causation for drought; they can just be not effective enough: they may capture the

pattern of changes, but perhaps not the magnitude. This is what we discuss in the later parts of the discussion.

Figures:
Fig 1 needs to show the data points – preferably using a boxplot approach. Or at minimum add 95%CI to show that these are significantly different. Looking at the variability in Fig 2 (estimates range from positive to negative), the standard errors seem smaller than is presented in Fig 2.

R.33. For the sake of clarity regarding our main message (i.e. the significant difference of mean effect size of the two study types), we would prefer to stick to the original type of graph in Fig. 1. We also assume that the data points of the individual effect sizes can be seen in both Fig. 2a and 2b, so it would be redundant to show them also on Figure 1. However, we changed the standard error bars into confidence intervals (CI) in Fig 1 as requested. Note that mean values have changed on the figure, because the actual means were replaced by the modelled ones (see R.23).

Fig 2 also needs 95%CI around the two lines at minimum.

R.33. We also added CI bands to both parts of Figure 2. Although CI bands are separated only in the middle part of Figure 2, please note that while separated CIs imply significant differences, overlapping confidence intervals do not necessarily mean the absence of significant difference between the groups; for further justification please see for example Krzywinski & Altman in Nature Methods (2013).

Editorial comments:
1. Lines 67-68: "large differences" is relative and unclear -- be specific.

R.34. We agree that the wording „large" hints that the size of the difference is important, without providing the specific detail. Thinking this over, we think that it is unnecessary to provide the specific number; the message here is that there is difference. So we changed the wording to „clear difference".

2. Lines 70 -71: There are contrasting and contradictory drought forecasts – cite a specific study or speak to this nuance.

R.35. We now provided two references for this statement (Trenberth et al. 2014, Cook et al. 2020).

3. Lines 95-98: restructure lengthy sentence

R.36. We now restructured and cut it into two separate sentences.

„In our focal meta-analysis, we weighted data by the number of replications (see Methods and ESM 6). We also conducted additional meta-analyses with different weightings, and using the data that were excluded from the focal analysis, to test the robustness of our results."

4. Line 102: "was unaffected by drought length (ESM 5)" reword, as non-significance ≠ unaffected

R.37. We reworded this part as follows:

„was not significantly affected by drought length"

Reviewer #3 (Remarks to the Author):

The paper by Kröel-Dulay and Mojzes et al. asks whether analysing the effects of drought on terrestrial plants leads to different results when relying on observation or experimentation. To answer this question they meta-analyse across different study types and take a range of potentially important covariates into account. They conclude that experiments systematically underestimate drought effects.

Being not a terrestrial ecologist I was asked to especially have a look on the conduction of the meta-analysis in this manuscript. I am very positive about the handling and reporting of the search, data handling, statistics and sensitivity analyses. The authors exemplarily follow the guidelines for reproducible synthesis and whenever I thought about a potential analyses needed to test for the sensitivity of decisions made, I found these in the supporting material.

R.38. We thank the Reviewer for the careful evaluation, and the positive opinion on the way we performed the data analysis. Please find our responses to the comments/questions below.

Still I have a few comments that need to be addressed.

1) Why only aboveground biomass or NPP? I am pretty sure that other response variables have been measured frequently in these experiments and it would be very informative to see

whether the observed differences are general across responses – is it just ANPP or are other processes less sensitive to the experimentation.

R.39. This, indeed, would be a very interesting question. We used aboveground biomass and ANPP because we wanted to include the highest number of studies for in-depth analyses on the same response variable. Aboveground biomass and NPP are the most widely used measures of ecosystem functioning. This is what most case studies report, and what synthesis studies most frequently focus on. Other variables, such as belowground biomass and soil respiration, are reported in much fewer studies than aboveground biomass or NPP. As an example, species richness, which is also considered to be an important characteristic of plant communities, could be found only in 20% of the papers in our focal data set. Thus, though we would have indeed preferred to include other relevant measures of ecosystem functioning in our analyses, we were restricted by the availability of data and had to focus on aboveground biomass and NPP to retain enough data for meaningful comparisons.

2) I agree that the size of the treatment application is a potential cause here. Drought experiments dry a small area of soil in a wet surrounding, whereas real droughts dry up larger regions. Thus, lateral flows of water and edge effects might not at all be important in observations, but surely are in the m^2 range of experimental plots. While this is somewhat discussed in the paper I wondered i) why it is not be made more explicit (row 148ff talks about a lot of differences, but does not make the main difference very clear) and ii) if there would not be a way to directly test this - at least experiments of different size must exist and it may be possible to also estimate the spatial extent of observed droughts.

R.40. Thank you for this suggestion. Indeed, this could be tested, but only for experimental studies as there are no data on the size of natural drought events, at least not in the papers that report drought effects. Also, natural droughts most likely do not have a clear boundary; they may just spatially vary in severity. However, drought experiments have a definite size, and papers usually report these data. We therefore gathered these data from the experimental studies of the focal data set, and tested in a separate meta-analysis, if effect size of aboveground biomass response is related to treatment size (the size of the drought treatment). We found no relationship (ESM 13), which we interprete as experiments inherently covering a too small range in size:

„Although we did not find a relationship between the size of drought experiments and the effect size of aboveground biomass response to drought in our dataset (ESM 13), even the size

of largest experiments (few studies were > 100 m^2) was extremely small compared to the spatial extent of natural drought events."

3) The authors argue, and bring some evidence, that experiments might underestimate the magnitude of global change effects. However, this is less general than stated here. Experiments tend to overdo the differences between treatments and control (see. E.g. Korell et al. 2020, Global Change Ecology) and have secondary crowding effects (Moise & Henry 2010 Oikos, Hillebrand 2009 J Phycol). The Korell paper is so close to the topic here that it should be included in the discussion. Moreover, I would like to see the discussion more nuanced and shifted towards why drought experiments may be especially prone to this underestimation.

R.41. Yes, there are examples when treatment effects are magnified in the small areas of the experiment, but these are usually secondary effects and act at higher trophic levels. We now mention this in the discussion:

„ Note that the island effect may also sometimes strengthen the treatment effect in experiments, but this usually happens as a secondary effect due to altered primary production or species composition, such as congregation or avoidance of animals"

Although the Korell paper emphasises that a priori set drought severity (and not treatment effect) is stronger in experiments compared natural droughts, and we accounted for drought severity, it is still a closely related paper, so we included it in the discussion:

„It has been suggested that experiments tend to exaggerate drought severity relative to natural droughts[16]. However, we found that drought severity was similar across experimental and observational studies, and we used an analysis that accounted for drought severity."

As we do not know the other research areas very well, we do not have a clear opinion on whether drought experiments would or would not be more prone for underestimation compared to other areas of environmental change. However, when we looked at the relevant papers form these other research areas, we found that the issues raised by the authors to explain weaker responses in experiments were similar to what we concluded for drought:

„Mechanisms suggested for these patterns were the same as those that may explain the differential drought effects in our study, namely the small spatial extent[21] and incomplete representation of environmental change factors in experiments[18,20]."

Please note that Brief Communications is a concise article format, which does not allow for an elaborate discussion (we are already beyond the suggested length). This prevented us from discussing our findings at length, in general, and also addressing the issues raised in your comment in detail. However, we agree that they are important and tried to include them, albeit briefly.

Decision Letter, first revision:

21st December 2021

Dear Dr. Kröel-Dulay,

Thank you for submitting your revised manuscript "Field experiments underestimate aboveground biomass response to drought" (NATECOLEVOL-210814292A). It has now been seen again by the original reviewers and their comments are below. The reviewers find that the paper has improved in revision, and therefore we'll be happy in principle to publish it in Nature Ecology & Evolution, pending minor revisions to comply with our editorial and formatting guidelines.

If the current version of your manuscript is in a PDF format, please email us a copy of the file in an editable format (Microsoft Word or LaTex)-- we can not proceed with PDFs at this stage.

We are now performing detailed checks on your paper and will send you a checklist detailing our editorial and formatting requirements in about a week (though please note it might be slightly longer than this given the upcoming holiday). Please do not upload the final materials and make any revisions until you receive this additional information from us.

Thank you again for your interest in Nature Ecology & Evolution. Please do not hesitate to contact me if you have any questions.

[REDACTED]

Reviewer #1 (Remarks to the Author):

This is the second manuscript version of "Field experiments underestimate aboveground biomass response to drought" that I am reviewing. I was generally positive about the original manuscript, although I had a few concerns related to the interpretation regarding underestimation of effects in experimental studies vs overestimation of effects in observational studies, the ecological expectations in the introduction and the novelty of the study. I am very happy to see that the authors took my concerns very serious and did a good job on responding to them / changing the new manuscript accordingly. Hence, I have no further comments.

Reviewer #2 (Remarks to the Author):

I thank the authors for their thoughtful responses. They have carefully considered each of my concerns, and while a few of them remain, the authors have identified these areas as important next steps or argued their perspectives effectively. Excellent job and congratulations on an important contribution to the literature.

Reviewer 3 - provided only confidential comments to the editor, indicating that they were satisified with the revision.

Our ref: NATECOLEVOL-210814292A

31st December 2021

Dear Dr. Kröel-Dulay,

Thank you for your patience as we've prepared the guidelines for final submission of your Nature Ecology & Evolution manuscript, "Field experiments underestimate aboveground biomass response to drought" (NATECOLEVOL-210814292A). Please carefully follow the step-by-step instructions provided in the attached file, and add a response in each row of the table to indicate the changes that you have made. Please also check and comment on any additional marked-up edits we have proposed within the text. Ensuring that each point is addressed will help to ensure that your revised manuscript can be swiftly handed over to our production team.

We would like to start working on your revised paper, with all of the requested files and forms, as soon as possible (preferably within two weeks). Please get in contact with us immediately if you anticipate it taking more than two weeks to submit these revised files.

When you upload your final materials, please include a point-by-point response to any remaining reviewer comments.

If you have not done so already, please alert us to any related manuscripts from your group that are under consideration or in press at other journals, or are being written up for submission to other journals (see: https://www.nature.com/nature-research/editorial-policies/plagiarism#policy-on-duplicate-publication for details).

In recognition of the time and expertise our reviewers provide to Nature Ecology & Evolution's editorial process, we would like to formally acknowledge their contribution to the external peer review of your manuscript entitled "Field experiments underestimate aboveground biomass response to drought". For those reviewers who give their assent, we will be publishing their names alongside the published

article.

Nature Ecology & Evolution offers a Transparent Peer Review option for new original research manuscripts submitted after December 1st, 2019. As part of this initiative, we encourage our authors to support increased transparency into the peer review process by agreeing to have the reviewer comments, author rebuttal letters, and editorial decision letters published as a Supplementary item. When you submit your final files please clearly state in your cover letter whether or not you would like to participate in this initiative. Please note that failure to state your preference will result in delays in accepting your manuscript for publication.

Cover suggestions

As you prepare your final files we encourage you to consider whether you have any images or illustrations that may be appropriate for use on the cover of Nature Ecology & Evolution.

Covers should be both aesthetically appealing and scientifically relevant, and should be supplied at the best quality available. Due to the prominence of these images, we do not generally select images featuring faces, children, text, graphs, schematic drawings, or collages on our covers.

We accept TIFF, JPEG, PNG or PSD file formats (a layered PSD file would be ideal), and the image should be at least 300ppi resolution (preferably 600-1200 ppi), in CMYK colour mode.

If your image is selected, we may also use it on the journal website as a banner image, and may need to make artistic alterations to fit our journal style.

Please submit your suggestions, clearly labeled, along with your final files. We'll be in touch if more information is needed.

Nature Ecology & Evolution has now transitioned to a unified Rights Collection system which will allow our Author Services team to quickly and easily collect the rights and permissions required to publish your work. Approximately 10 days after your paper is formally accepted, you will receive an email in providing you with a link to complete the grant of rights. If your paper is eligible for Open Access, our Author Services team will also be in touch regarding any additional information that may be required to arrange payment for your article.

Please note that <i>Nature Ecology & Evolution</i> is a Transformative Journal (TJ). Authors may publish their research with us through the traditional subscription access route or make their paper immediately open access through payment of an article-processing charge (APC). Authors will not be required to make a final decision about access to their article until it has been accepted. Find out more about Transformative Journals

<B>Authors may need to take specific actions to achieve compliance with funder and institutional open access mandates. For submissions from January 2021, if your research is supported by a funder that requires immediate open access (e.g. according to Plan S

principles) then you should select the gold OA route, and we will direct you to the compliant route where possible. For authors selecting the subscription publication route our standard licensing terms will need to be accepted, including our self-archiving policies. Those standard licensing terms will supersede any other terms that the author or any third party may assert apply to any version of the manuscript.

Please note that you will not receive your proofs until the publishing agreement has been received through our system.

For information regarding our different publishing models please see our Transformative Journals page. If you have any questions about costs, Open Access requirements, or our legal forms, please contact ASJournals@springernature.com.

Please use the following link for uploading these materials:
[REDACTED]

If you have any further questions, please feel free to contact me.

[REDACTED]

Reviewer #1:
Remarks to the Author:
This is the second manuscript version of "Field experiments underestimate aboveground biomass response to drought" that I am reviewing. I was generally positive about the original manuscript, although I had a few concerns related to the interpretation regarding underestimation of effects in experimental studies vs overestimation of effects in observational studies, the ecological expectations in the introduction and the novelty of the study. I am very happy to see that the authors took my concerns very serious and did a good job on responding to them / changing the new manuscript accordingly. Hence, I have no further comments.

Reviewer #2:
Remarks to the Author:
I thank the authors for their thoughtful responses. They have carefully considered each of my concerns, and while a few of them remain, the authors have identified these areas as important next steps or argued their perspectives effectively. Excellent job and congratulations on an important contribution to the literature.

Reviewer #3:
None

Final Decision Letter:

27th January 2022

Dear Dr Kröel-Dulay,

We are pleased to inform you that your Brief Communication entitled "Field experiments underestimate aboveground biomass response to drought", has now been accepted for publication in Nature Ecology & Evolution.

Over the next few weeks, your paper will be copyedited to ensure that it conforms to Nature Ecology and Evolution style. Once your paper is typeset, you will receive an email with a link to choose the appropriate publishing options for your paper and our Author Services team will be in touch regarding any additional information that may be required

After the grant of rights is completed, you will receive a link to your electronic proof via email with a request to make any corrections within 48 hours. If, when you receive your proof, you cannot meet this deadline, please inform us at rjsproduction@springernature.com immediately.

You will not receive your proofs until the publishing agreement has been received through our system

Due to the importance of these deadlines, we ask you please us know now whether you will be difficult to contact over the next month. If this is the case, we ask you provide us with the contact information (email, phone and fax) of someone who will be able to check the proofs on your behalf, and who will be available to address any last-minute problems . Once your paper has been scheduled for online publication, the Nature press office will be in touch to confirm the details.

Acceptance of your manuscript is conditional on all authors' agreement with our publication policies (see www.nature.com/authors/policies/index.html). In particular your manuscript must not be published elsewhere and there must be no announcement of the work to any media outlet until the publication date (the day on which it is uploaded onto our web site).

Please note that <i>Nature Ecology & Evolution</i> is a Transformative Journal (TJ). Authors may publish their research with us through the traditional subscription access route or make their paper immediately open access through payment of an article-processing charge (APC). Authors will not be required to make a final decision about access to their article until it has been accepted. Find out more about Transformative Journals

<B>Authors may need to take specific actions to achieve compliance with funder and institutional open access mandates. For submissions from January 2021, if your research is supported by a funder that requires immediate open access (e.g. according to Plan S

principles) then you should select the gold OA route, and we will direct you to the compliant route where possible. For authors selecting the subscription publication route our standard licensing terms will need to be accepted, including our self-archiving policies. Those standard licensing terms will supersede any other terms that the author or any third party may assert apply to any version of the manuscript.

In approximately 10 business days you will receive an email with a link to choose the appropriate publishing options for your paper and our Author Services team will be in touch regarding any additional information that may be required.

You will not receive your proofs until the publishing agreement has been received through our system.

If you have any questions about our publishing options, costs, Open Access requirements, or our legal forms, please contact ASJournals@springernature.com

An online order form for reprints of your paper is available at https://www.nature.com/reprints/author-reprints.html. All co-authors, authors' institutions and authors' funding agencies can order reprints using the form appropriate to their geographical region.

We welcome the submission of potential cover material (including a short caption of around 40 words) related to your manuscript; suggestions should be sent to Nature Ecology & Evolution as electronic files (the image should be 300 dpi at 210 x 297 mm in either TIFF or JPEG format). Please note that such pictures should be selected more for their aesthetic appeal than for their scientific content, and that colour images work better than black and white or grayscale images. Please do not try to design a cover with the Nature Ecology & Evolution logo etc., and please do not submit composites of images related to your work. I am sure you will understand that we cannot make any promise as to whether any of your suggestions might be selected for the cover of the journal.

You can now use a single sign-on for all your accounts, view the status of all your manuscript submissions and reviews, access usage statistics for your published articles and download a record of your refereeing activity for the Nature journals.

To assist our authors in disseminating their research to the broader community, our SharedIt initiative provides you with a unique shareable link that will allow anyone (with or without a subscription) to read the published article. Recipients of the link with a subscription will also be able to download and print the PDF.

You can generate the link yourself when you receive your article DOI by entering it here: <a href="http://authors.springernature.com/share">http://authors.springernature.com/share<a>.

[REDACTED]

P.S. Click on the following link if you would like to recommend Nature Ecology & Evolution to your librarian http://www.nature.com/subscriptions/recommend.html#forms

** Visit the Springer Nature Editorial and Publishing website at www.springernature.com/editorial-and-publishing-jobs for more information about our career opportunities. If you have any questions please click here.**
